# Assembly Processes and Co-occurrence Patterns of Abundant and Rare Bacterial Community in the Eastern Indian Ocean

**DOI:** 10.3389/fmicb.2021.616956

**Published:** 2021-08-11

**Authors:** Liuyang Li, Laxman Pujari, Chao Wu, Danyue Huang, Yuqiu Wei, Congcong Guo, Guicheng Zhang, Wenzhe Xu, Haijiao Liu, Xingzhou Wang, Min Wang, Jun Sun

**Affiliations:** ^1^Research Centre for Indian Ocean Ecosystem, Tianjin University of Science and Technology, Tianjin, China; ^2^School of Life Sciences and Biotechnology, Shanghai Jiao Tong University, Shanghai, China; ^3^Institute of Marine Science and Technology, Shandong University, Qingdao, China; ^4^College of Biotechnology, Tianjin University of Science and Technology, Tianjin, China; ^5^College of Marine Life Sciences, Institute of Evolution and Marine Biodiversity, Ocean University of China, Qingdao, China; ^6^College of Marine Science and Technology, China University of Geosciences, Wuhan, China

**Keywords:** bacterial community, community assembly, species coexistence, null model, rare taxa, abundant taxa, Eastern Indian Ocean

## Abstract

Microbial communities are composed of many rare species and a few abundant species. Considering the disproportionate importance of rare species for ecosystem functioning, it is important to understand the mechanisms structuring the rare and abundant components of a diverse community in response to environmental changes. Here, we used a 16S ribosomal RNA gene sequencing approach to investigate the bacterial community diversity in the Eastern Indian Ocean (EIO) during the monsoon and intermonsoon. We employed a phylogenetic null model and network analysis to evaluate the assembly processes and co-occurrence pattern of the microbial community. We found that higher bacterial diversity was detected in the intermonsoon with high temperature and low Chlorophyll *a* concentrations and N/P ratios. The balance between ecological deterministic processes and stochastic processes varied with seasons in the EIO. Meanwhile, conditionally rare taxa (CRT) were more likely modulated by variable selection processes than always rare taxa (ART) and abundant taxa (AT) (CRT > ART > AT). By linking assembly process and species co-occurrence, we demonstrated that the microbial co-occurrence associations tended to be higher when deterministic processes (mainly variable selection) were weaker. This negative trend was observed in rare species rather than abundant species. The linkage could enhance our understanding of the underlying mechanisms underpinning the generation and maintenance of microbial community diversity.

## Introduction

Constructing the linkage between community assembly and species co-occurring is important in ecology. In a local community, the assembly processes are divided into two types, ecologists naming them niche and neutral process (Stegen et al., 2012; Wang et al., 2013; Dini-Andreote et al., 2015; Tripathi et al., 2018). Niche theory assumes that microbial communities are shaped by deterministic processes consisting of variable selection and homogeneous selection, owing to different habitat preferences and fitness of species (Fargione et al., 2003; Bahram et al., 2016). In contrast, neutral theory holds that all members in microbial communities are ecologically functionally equivalent, and communities are shaped by stochastic processes involving dispersal limitation and homogenizing dispersal, as well as the undominated process consisting of weak selection, weak dispersal, and diversification or drift (Chave, 2004; Bahram et al., 2016; Zhou and Ning, 2017; Tripathi et al., 2018). These ecological processes can drive community assembly concurrently (Fargione et al., 2003; Langenheder and Szekely, 2011; Zhou and Ning, 2017; Jiao and Lu, 2020a). Coexistence theories can also be divided into two categories: deterministic niche partitioning in space of distinct ecotypes (Silvertown, 2004) and stochastic dispersal or demographic processes (Hubbell, 2001; Konopka et al., 2015). Examining of the relative importance of deterministic and stochastic processes in community assembly can illustrate the ecological strategies of co-occurring species (Kraft et al., 2015). Meanwhile, a complex ecological association network could further reveal the intrinsic mechanisms of microbial interactions following environmental changes (Hunt and Ward, 2015). However, natural systems are highly complex (Hooper et al., 2005), and disentangling the relative contributions of each ecological process in ecosystem is challenging (Dini-Andreote et al., 2015; Evans et al., 2017; Jiao et al., 2020).

Microbial communities are composed of many rare species with a few individuals and some abundant species with countless individuals (Logares et al., 2014). Accounting for most of the biomass and carbon cycling (Pedros-Alio, 2012), abundant taxa (AT) have been studied comprehensively to understand their key ecological functions. However, conditionally rare taxa (CRT), which are usually rare but occasionally become prominent in suitable conditions, have been reported to help ecologists identify the biological, chemical, and physical drivers of microbial dynamic in ecosystems (Shade et al., 2014; Chen et al., 2019). Although rare taxa (RT) are often referred to as the “rare biosphere” (Sogin et al., 2006; Pedros-Alio, 2012), they might have stronger environmental adaptation than AT in some environments (Wan et al., 2021a). Meanwhile, RT may be a hidden driver of ecological function with a disproportional role in biogeochemical cycles (Jousset et al., 2017). Several research have unveiled the unique functional role of rare species, for instance, fulfilling essential functions associated with nutrient cycling, phenanthrene degradation and their ecological importance, and as keystone species in regulating the functioning of aquatic environments (Elshahed et al., 2008; Pester et al., 2010; Sauret et al., 2014; Shade et al., 2014; Lynch and Neufeld, 2015; Xue et al., 2018; Chen et al., 2019; Jiao and Lu, 2020a). Moreover, abundant and rare community assemblages are probably influenced by different assembly processes (Xue et al., 2018). Given the ecological importance of rare taxa, a comprehensive comparison of them with AT in constructing the linkage between community assembly and species co-occurrence will help us to have a deeper understanding of microbial communities (Pedros-Alio, 2012; Lynch and Neufeld, 2015).

In the marine environment, little is known about fundamental processes governing the abundant and rare microbial community assembly, as well as the coexistence mechanisms (Wu et al., 2017). It has been recently reported that homogeneous selection accounts for 76.69–100% of the assembly processes in the temporally stable oligotrophic South Pacific Gyre, where bacterial communities are compositionally similar consisting of *Prochlorococcus* and SAR11 (Allen et al., 2020). In addition, in coastal sediments, dispersal limitation explains ∼40% of the community variation, structuring benthic archaeal communities characterized with Marine Group I (MG-I) of *Thaumarchaeota* and *Woesearchaeota* (Liu et al., 2020). These studies indicate that the assembly processes of the microbial community are not conserved between different marine ecosystems, and their correlations with species coexistence under changing environments are still a mystery.

As one of the largest oligotrophic areas, the Indian Ocean plays an essential role in global climatic change, material cycles, and energy flow (Schott et al., 2009). Winds and currents over the Eastern Indian Ocean (EIO) are seasonally reversed during the southwest monsoon (June–September) and northeast monsoon (October–February) with a clear transition phase, namely, the intermonsoon (March–May) (Jyothibabu et al., 2017). In monsoon, upwelling and convective mixing driven by seasonal monsoon winds could increase upward transportation of nutrients, resulting in seasonal blooms of phytoplankton (Lévy et al., 2007; Singh and Ramesh, 2015; Pujari et al., 2019). On the contrary, intermonsoon offered an extremely oligotrophic condition with higher temperature, since the minor disturbance and highly stratified water results in low supplement of the nutrients from deeper water (Wu et al., 2019). This seasonal oceanic region is thus an ideal place to test the linkage between community assembly and species co-occurrence under changing environments.

Here, we investigated the seasonal patterns of bacterial community in the EIO using 16S rRNA gene-based high-throughput sequencing. The aims of the present study were (I) to evaluate the relative importance of deterministic and stochastic processes in shaping rare and abundant microbial communities across different seasons in EIO, and (II) to establish the linkages between community assembly and species co-occurrence for total, rare, and abundant microbial communities under different environments.

## Materials and Methods

### Sample Collection and Environmental Parameter Analysis

Sampling was conducted during the 2016 EIO monsoon cruise (November–December) and 2017 EIO intermonsoon cruise (March–April), and surface sea water (5 m) was collected at 24 stations. Given that the spatial distribution of the samples from the two seasons was not strictly matched (Supplementary Figure 1), we carried out rigorous statistical verification to confirm that the diversity of bacterial communities were undoubtedly controlled by seasonal variation, and only little influence was introduced by spatial deviation (Supporting Information). Samples were collected using 5-L bottles attached to a rosette multisampler, on which conductivity, temperature, and depth (CTD) probes were installed (Seabird SBE 911plus, Sea-Bird Electronics, Washington, United States). To obtain the material for 16S rRNA gene analysis, about 2–4 L of surface water were filtered through a 0.22-μm-pore-size filter (47 mm in diameter, Millipore, Eschborn, Germany) with low pressure (more than −0.03 Mp). The filters were flash-frozen in liquid nitrogen on board and then transferred into a −80°C refrigerator upon returning to the laboratory until DNA extraction.

Surface water for inorganic nutrients were subsampled into 100-ml PE bottles that were previously rinsed with 10% HCl and Milli-Q water successively. Subsequently, the PE bottles were stored at 4°C until laboratory analysis. The concentrations of nutrients consisting of nitrate (NO_3_^–^), nitrite (NO_2_^–^), ammonium (NH_4_^+^), silicate (SiO_3_^2–^), and phosphate (PO_4_^3–^) were measured based on the classical colorimetric method (Grasshoff et al., 2009) with a Technicon AA3 AutoAnalyzer (Bran + Luebbe, Norderstedt, Germany). In brief, the principles of measurement for NO_3_^–^ or NO_2_^–^, NH_4_^+^, PO_4_^3–^, and SiO_3_^2–^ are the copper–cadmium column reduction method, indophenol blue method, silico-molybdate complex method, and phosphor-molybdate complex method, respectively.

Other subsamples for Chlorophyll *a* (Chl *a*) analysis were collected by vacuum-filtering 500 ml of water through a 0.7-μm-pore-size GF/F filter (25 mm in diameter, Waterman, Florham Park, NJ, United States) under a pressure of more than -10 mmHg. The filters were subsequently placed in aluminum foil paper bags and stored promptly in darkness at −20°C. In the laboratory, Chl *a* were extracted under dark conditions with 90% acetone and then determined using a Turner-Designs Trilogy^®^ fluorometer (San Jose, CA, United States). Other physicochemical parameters including temperature and salinity were measured and recorded by a CTD rosette system on board (Seabird SBE 911plus, Sea-Bird Electronics, Washington, United States).

### DNA Extraction and Sequencing

Extraction and purification of DNA were carried out using the DNeasy PowerWater^®^ Kit (Qiagen, Hilden, Germany) following the manufacturer’s protocol. An ND-2000 Nanodrop spectrometer (Thermal Scientific, Wilmington, DE, United States) was used to examine the quality and quantity of the extracted DNA. The V3–V4 region of the prokaryotic small-subunit 16S rRNA gene was amplified using the pairwise common primer 338F (5′-ACTCCTACGGGAGGCAGCAG-3′) and 806R (5′-GGACTACHVGGGTWTCTAAT-3′) (Mori et al., 2013). The PCR mixture contained 12.5 μl of 2× Taq PCR MasterMix, 3 μl of BSA (2 ng/μl), 1 μl of each primer (5 μM), and ∼30 ng of sample DNA, and the rest was nuclease-free water that was added to a final volume at 25 μl. The PCR reaction conditions were denatured (95°C, 5 min), followed by 28 cycles of denaturation (95°C, 45 s), annealing (55°C, 50 s), and extension (72°C, 45 s) with a final extension (72°C, 10 min). The triplicate PCR products for each sample were pooled in equal quantity and purified through the MinElute^®^ PCR Purification Kit (Qiagen, Hilden, Germany). The purified products were validated by Nanodrop 2000 and sequenced on the Illumina MiSeq PE300 platform (Illumina, Inc., San Diego, CA, United States) *via* paired-end chemistry.

### Quality Control and Sequencing Data Processing

According to the specific barcode sequence, the raw sequences were identified and separated by independent samples under the limit of no more than one mismatch. The barcode and adapter sequences were eliminated when the data were stored. An open-source bioinformatics pipeline QIIME was used to analyze and process 16S rRNA gene sequences preliminarily (Caporaso et al., 2010). The paired-end sequences were merged by FLASH (v1.2.7) software (Magoc and Salzberg, 2011), and the generated raw tags were quality filtered through Trimmomatic (v0.33) software (Bolger et al., 2014). After that, high-quality clean tags were obtained without the barcodes, linker sequences, and primers. Furthermore, chimeras were removed to get effective tags from the above high-quality tags using the USEARCH (v10.0) tool based on the UCHIME algorithm (Edgar et al., 2011). To minimize sequencing errors, singletons were eliminated before the cluster of operational taxonomic units (OTUs). Subsequently, UPARSE pipeline was applied to cluster the remaining tags, among which sharing more than 97% similarity were assigned to the same OTUs (Edgar et al., 2011). Based on the RDP Classifier algorithm, representative sequences obtained from each OTU were aligned against the SILVA 128 reference. Sequence numbers were normalized and 20,446 sequences for each sample were finally generated. The ultimate total data set retained 3,047 OTUs at 97% similarity level.

### Definition of Rare Taxa and Abundant Taxa

We modified a previous definition (Dai et al., 2016; Chen et al., 2017) by classifying all OTUs into CRT, always rare taxa (ART), and AT. Briefly, this definition distinguishes OTUs artificially based on the cutoff level of their relative abundance at different samples by setting 0.01% as rare OTUs and 1% as abundant OTUs. CRT represent OTUs with relative abundance <1% in all samples and <0.01% in some samples, simultaneously. ART indicate OTUs with relative abundance <0.01% in all samples. In addition, AT are defined as the sum of always abundant taxa (AAT, relative abundance ≥1% in all samples), conditionally abundant taxa (CAT, relative abundance ≥0.01% in all samples and ≥1% in some samples, simultaneously), conditionally rare and abundant taxa (CRAT, relative abundance containing <0.01 and ≥1% taxa at different samples), and moderate taxa (MT, relative abundance between 0.01 and 1% in all samples). This simplified definition can strengthen our understanding of rare taxa (especially CRT) under different conditions compared with AT.

### Statistical Analysis

All statistical analyses were performed in R (v3.6.1^1^), using the “vegan” (Oksanen et al., 2015), “stats” (Field et al., 2012), “picante” (Kembel et al., 2010), “SoDA” (Vincenty, 2013), “Hmisc” (Harrell and Dupont, 2013), “igraph” (Csardi and Nepusz, 2006), “gbmplus” (De’ath, 2007), and “ggplot2” (Wickham, 2016) packages.

### Seasonal Variation Based on Community Diversity Indices

Alpha-diversity indices were calculated at the OTU level by using diversity function in the “vegan” package (Oksanen et al., 2015). Seasonal effect on alpha-diversity was examined by one-way analysis of variance (ANOVA). Bray–Curtis dissimilarities were calculated based on the vegdist function. In order to interpret the distribution patterns of the bacterial communities, non-metric multidimensional scaling (NMDS), and unweighted pair-group method with arithmetic means (UPGMA) were used to represent bacterial community characters based on Bray–Curtis dissimilarities.

### Community Assembly Process

To evaluate the relative importance of deterministic or stochastic processes in microbial communities, a null modeling approach was applied using the framework as Stegen et al. (2013) described. Before applying the null modeling approach, phylogenetic signal in OTUs’ optimal habitat conditions was detected following the procedure described previously (Stegen et al., 2013). Subsequently, the pairwise phylogenetic turnover (βMNTD) that quantifies the phylogenetic distance for each OTU was calculated using the “comdistnt” function in the “picante” package (Kembel et al., 2010). The degree to which βMNTD deviates from the turnover expected measures the selection effect controlled by OTU ecological niches. To quantify the degree to which βMNTD deviates from an expectation, β-nearest taxon index (βNTI) is introduced, which is the difference between observed βMNTD and mean of null expectation normalized by standard deviation of null expectation. βNTI for all pairwise comparisons was quantified using a separate null model repeating the randomization 999 times. | βNTI| > 2 indicates the dominance of deterministic processes, whereas | βNTI| < 2 indicates the influence of stochastic processes. In detail, βNTI > +2 indicates significantly greater phylogenetic turnover than null expectation, thus corresponding to variable selection, whereas βNTI < -2 indicates significantly less turnover than expectation, thus corresponding to homogeneous selection. If | βNTI| < 2, Bray–Curtis-based Raup–Crick (RC_*Bray*_) was extended to quantify stochastic processes (Chase et al., 2011; Stegen et al., 2013). RC_*Bray*_ > +0.95 indicates that the community assembly is governed by the dispersal limitation, whereas RC_*Bray*_ < -0.95 indicates the dominant homogenizing dispersal process. In addition, the fractions of remnant | βNTI| < 2 and | RC_*Bray*_| < 0.95 suggest the “undominated” community assembly, consisting of weak selection, weak dispersal, and diversification or drift.

To support the null model results, variation partitioning analysis (VPA) was applied to distinguish the relative contribution of environmental selection and dispersal effect in microbial community assembly by separating community variation (pairwise Bray–Curtis dissimilarity) into environmental and spatial effects (Yeh et al., 2015). Spatial components were obtained from principal coordinates analysis of neighbor matrices (PCNM) (Dray et al., 2006; Griffith and Peres-Neto, 2006). The derived PCNM variables (e.g., PCNM1 and PCNM2) were regarded as capturing all the detectable spatial scales (Dray et al., 2006; Griffith and Peres-Neto, 2006). This process was performed using the pcnm function in the “vegan” package (Oksanen et al., 2015), before which the latitude and longitude coordinates of the sampling points were converted into the Cartesian coordinate system by the geoXY function in the “SoDA” package (Vincenty, 2013). Based on a constrained analysis of the principal coordinates model, the derived *R*^2^ were adjusted using the RsquareAdj function (Peres-Neto et al., 2006; Legendre et al., 2011). Variance inflation factors (VIFs) were examined by co-linearity test conducted with the vif.cca function in the “vegan” package (Oksanen et al., 2015). Subsequently, forward selection procedures were performed using the ordiR2step function to select spatial and environmental variables. The procedures were ceased if the significance level (*p*-value) of the model exceeded 0.05. Independent spatial variation that excludes the environmental component represents the dispersal effect. Similarly, independent environmental variation without a spatial component corresponds to the effect of environmental selection.

### Co-occurrence Network Analysis

To estimate the seasonal pattern of species coexistence, metacommunity co-occurrence networks consisting of the monsoon and intermonsoon were constructed. To make the data sets computable, OTUs containing more than 20 sequences were retained for the construction of networks. A total of 947 OTUs were used to calculate pairwise Spearman’s rank correlations (*r*) within the “hmisc” R package (Harrell and Dupont, 2013). Only robust (*r* > 0.8 or *r* < -0.8) and statistically significant (*p* < 0.01) correlations were used in network analysis. Subsequently, node-level topological properties, including degree, betweenness, closeness, and eigenvector, were calculated in the “igraph” R package (Csardi and Nepusz, 2006). The values of these topological features reflect the roles of nodes in the network (high value representing a core position; low value representing a peripheral position). A set of network-level topological features, such as average degree, connectance, diameter, average path length, and clustering coefficient, were also calculated. The robustness of a network was tested using natural connectivity of a complex network (Jun et al., 2010). Networks were visualized based on Gephi v 0.9.2, and modular analysis was also performed to research the inner-network structure between the two seasons.

To identify significantly seasonal enriched taxa of bacterial communities, we applied Wilcoxon rank-sum test to the relative abundance of each OTU between monsoon and intermonsoon samples using the wilcox.test function in the “stats” package (Field et al., 2012). OTUs with significantly higher abundance (*p* < 0.05) in monsoon samples were categorized as monsoon-enriched OTUs, whereas those possessing significantly higher abundance (*p* < 0.05) in intermonsoon samples were grouped as intermonsoon-enriched OTUs, and the remaining OTUs corresponding to no significant differences in relative abundance were defined as “Others.”

### Drivers of Microbial Community

An aggregated boosted tree (ABT) was used to quantify the effect of environmental factors on the bacterial community diversity using the “gbmplus” package with 500 trees for boosting (De’ath, 2007). We implemented the Mantel test to select significant environmental variables correlated with the variations of microbial communities. In addition, to clarify the negative or positive effect, we correlated the dissimilarities of microbial communities and differences in the relative abundance of different phyla to the differences in environmental properties.

## Results

### Seasonal Variation of Bacterial Community Structure

A data set with 490,704 high-quality sequences and 3,047 OTUs was obtained. The flat trend of rarefaction curve indicated a reasonable amount of sequencing data (Supplementary Figure 1). Meanwhile, the truncated Preston log-normal model was used to estimate the expected community richness. Low Preston veil values suggested that adequate richness of the bacterial community was captured (96.49–96.67%).

**FIGURE 1:**
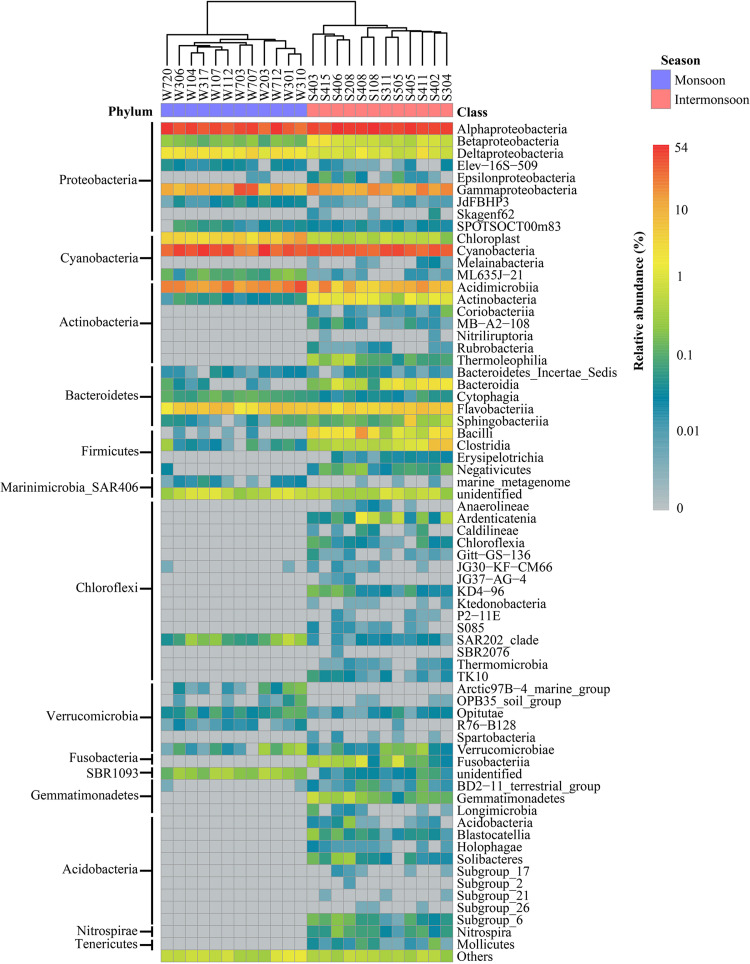
Bacterial community composition at class level in the Eastern India Ocean (EIO) in the monsoon and intermonsoon.

The taxonomic compositions of the bacterial community were different in the monsoon and intermonsoon (Figure 1). In general, 16 and 29 bacterial phyla were detected in the two seasons, respectively. The predominant phyla (≥1% of all sequences) of the monsoon bacterial community included Proteobacteria (average, 45.83%), Cyanobacteria (34.55%), Actinobacteria (14.49%), and Bacteroidetes (3.42%), together accounting for 98.29% of monsoon sequences, while the shift of bacterial community structure occurred in the intermonsoon, including an increase of dominant phyla Proteobacteria (53.94%), Bacteroidetes (4.31%), and Firmicutes (3.39%), and a decrease of Cyanobacteria (28.77%) and Actinobacteria (7.36%). It was notable that, in the intermonsoon samples, the percentages of Firmicutes ranging from 0.74 to 11.62% were in stark contrast with those in the monsoon samples whose maximum percentage was only 0.30% (*p* < 0.001). Proteobacteria, the most abundant phylum, mainly consisted of the following classes: Alphaproteobacteria (monsoon 33.73%, intermonsoon 42.34%), Gammaproteobacteria (10.08%, 9.92%), Deltaproteobacteria (1.70%, 0.83%), and Betaproteobacteria (0.15%, 0.75%). In addition, *Prochlorococcus* belonging to Cyanobacteria and SAR11 belonging to Alphaproteobacteria were observed to be the most AT.

### Seasonal Variation of Abundant and Rare Bacterial Community

A total of 2,968 OTUs (97.41%) representing 17.64% of all sequences were defined as RT, whereas 79 OTUs (2.59%) contributing 82.36% of all sequences were affiliated to AT (Supplementary Table 1). We found a significantly positive relationship between relative abundance and species occurrence (Supplementary Figure 2), suggesting that AT had wider niche breadths than RT. Majority OTUs belonging to AT (96.2%) were shared between the two seasons, while many members in RT were seasonal specific (56.9%) (Supplementary Figure 3). Significant increases in the Richness index, ACE index, and Chao1 index from the monsoon to intermonsoon were observed according to ANOVA analysis (Supplementary Figure 4). CRT contributed a large part to the seasonal variation of community diversity, since many species that were rarely detected in the monsoon emerged in the intermonsoon.

The NMDS and ANOSIM showed a strong seasonal pattern of bacterial communities, and CRT presented a stronger seasonal pattern compared with ART and AT (Supplementary Figure 5 and Supplementary Table 3). Both Bray–Curtis dissimilarity and environmental parameters had significant seasonal variations, indicating that microbial communities and local environmental conditions went through a dramatic change between seasons (Supplementary Figures 5, 6).

### Distinct Assembly Processes of Bacterial Community in the Monsoon and Intermonsoon

**FIGURE 2:**
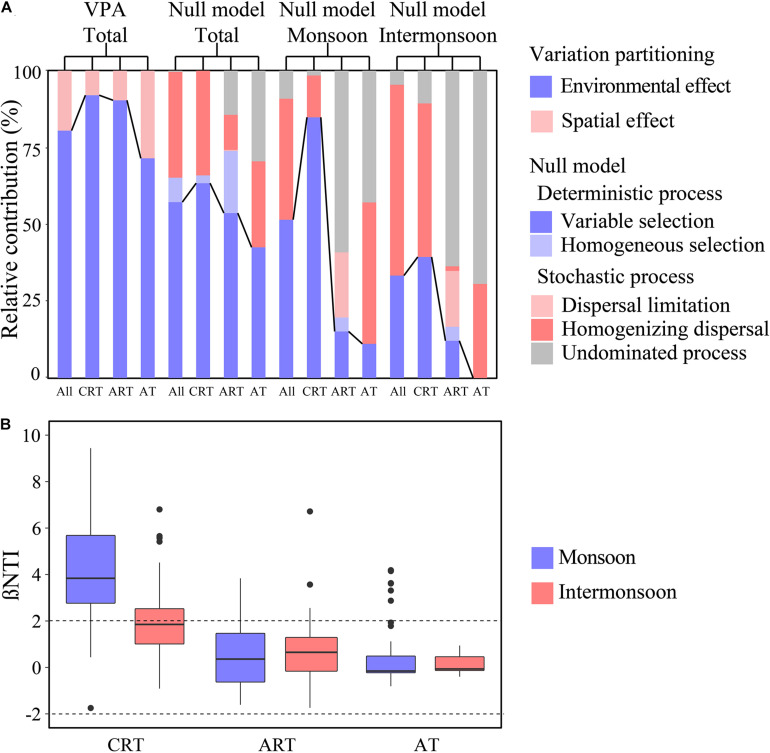
Community assembly mechanisms for different taxa in the monsoon and intermonsoon. **(A)** The assembly processes of bacterial community in the EIO. Null model: community assembly processes were governed primarily by deterministic processes, including variable and homogeneous selection, and stochastic processes, consisting of dispersal limitations and homogenizing dispersal, as well as the undominated process that was not dominated by any single process. Variation partitioning analysis (VPA) depicting the relative contribution of environmental and spatial effects on microbial community assembly. **(B)** The seasonal patterns of βNTI for CRT, ART, and AT. CRT, conditionally rare taxa; ART, always rare taxa; AT, abundant taxa.

We detected a significant phylogenetic signal across short phylogenetic distances in OTU environmental niches (Supplementary Figure 7), indicating that ecological processes inferred based on phylogenetic turnover were reliable. A large part of | βNTI| values were higher than 2, indicating that the effect of the deterministic process was more important than the stochastic process in the total community (Figure 2), while in the intermonsoon, the importance of stochasticity overwhelmed the determinism. Furthermore, there was a high proportion of variable selection (57.2%) and homogenizing dispersal (34.4%) in the total community, while homogeneous selection was detected with a little proportion (8.0%). Variable selection processes (51.5%) seemed to be more important than homogenizing dispersal (39.4%) for bacterial community in the monsoon, and a relatively higher proportion of homogenizing dispersal processes was estimated in the intermonsoon (62.1%). We have not detected dispersal limitation using the null model. In addition, compared with the significant influence of environmental effects, little spatial effects influencing the microbial community were calculated according to VPA.

### Seasonal Dynamics of Co-occurrence Network

**FIGURE 3:**
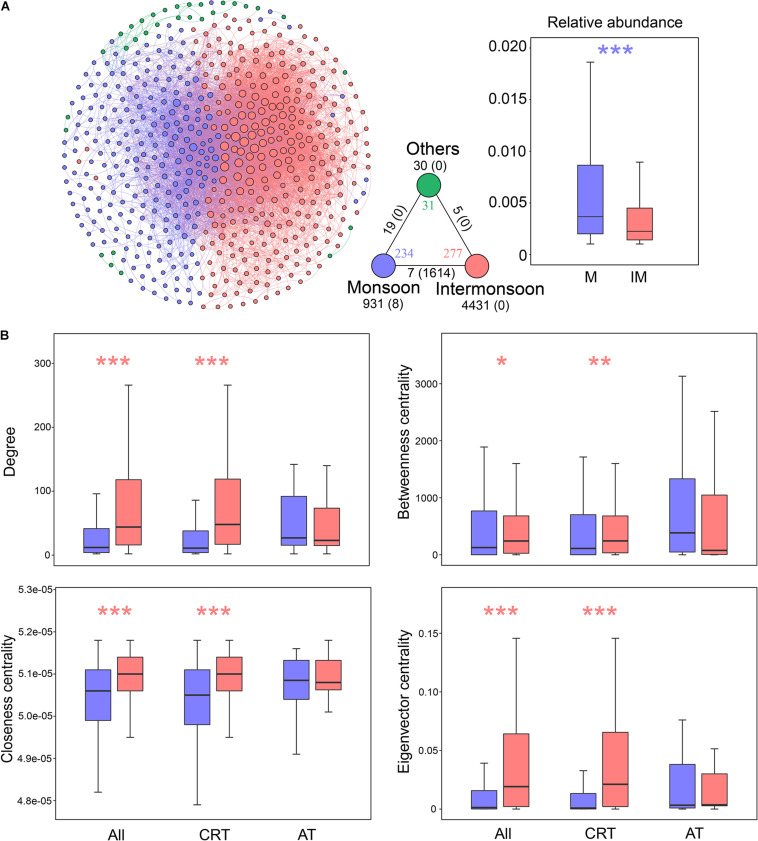
Properties of the co-occurrence network in bacterial communities during the monsoon and intermonsoon periods. **(A)** The network was conducted by strong (Spearman’s | *r*| > 0.8) and significant (*p*-value < 0.01) correlations between OTUs in the metacommunity. Each node represents an OTU, and each edge represents a correlation. The size of nodes is proportional to the degree of corresponding OTUs (the number of connections). The networks were colored based on monsoon or intermonsoon enriched OTUs, showing the intra-associations within each season and inter-associations between the two seasons. The triangle located at the bottom right of the network figure is a summary of the intra-associations and inter-associations. Colored numbers indicate the number of nodes belonging to the corresponding category (i.e., there were 234 nodes in the “monsoon” category). Numbers outside and inside parentheses, respectively, indicate the numbers of positive edges and negative edges. Black numbers near the apex of the triangle in turn indicate the number of intra-associations. Numbers near the triangle edge in turn indicate the number of cross-group interactions. The boxplot showing the relative abundance of seasonal enriched OTUs. **(B)** Seasonal comparison of node-level topological features among metacommunity and subcommunities. Blue asterisks indicate the values significantly higher in monsoon-enriched taxa (*p < 0.05; **p < 0.01; ***p < 0.001; Wilcoxon rank-sum test); red asterisks indicate the properties significantly higher in intermonsoon-enriched taxa. Monsoon, monsoon-enriched OTUs; Intermonsoon, intermonsoon-enriched OTUs; Others, OTUs not specific to a group. RT, rare taxa; AT, abundant taxa.

We inferred a co-occurrence network based on correlation relationships. A total of 542 nodes and 7045 edges were generated, which, respectively, represent OTUs and significant correlations between two OTUs. The co-occurrence network was mainly structured by seasonally enriched OTUs, consisting of 234 monsoon-enriched OTUs and 277 intermonsoon-enriched OTUs (Figure 3 and Supplementary Table 5). The unique node-level topological features of intermonsoon-enriched OTUs, such as degree, betweenness, closeness, and eigenvector centrality, were significantly higher than those of monsoon-enriched OTUs (Figure 3B). The monsoon network included 931 positive edges and 8 negative edges, while the intermonsoon network contained 4,431 positive edges without negative edges. There were 1,614 negative edges between monsoon- and intermonsoon-enriched OTUs, indicating potential competitions and/or turnover among bacterial species.

We further generated subnetworks for monsoon- and intermonsoon-enriched communities and calculated a set of network-level topological features (Supplementary Table 5). All the networks exhibited scale-free characteristics (Supplementary Figure 8), as suggested by *R*^2^ of power law (0.965, 0.939, and 0.742 for the total community, the monsoon community, and the intermonsoon community, respectively). The average path length and average clustering coefficient of real networks were greater than those of their respective random networks generated with identical numbers of nodes and edges, indicating that these networks had “small-world” properties (Supplementary Table 5). The average degree, connectance, and clustering coefficient were higher in the intermonsoon subnetwork than in the monsoon subnetwork, suggesting that intermonsoon-enriched OTUs were more interconnected. The diameter and average path length were lower in the intermonsoon subnetwork, revealing closer relationships among intermonsoon-enriched communities. These results indicated that species co-occurred more frequently within the intermonsoon bacterial communities than within the monsoon bacterial communities.

Subsequently, we estimated the network robustness by removing nodes from season-enriched communities (Supplementary Figure 8). The natural connectivity of the intermonsoon-enriched communities was higher than the monsoon-enriched communities, suggesting that microbial communities in the intermonsoon were more robust. However, monsoon-enriched communities were highly modular compared with intermonsoon-enriched communities (Supplementary Figure 9 and Supplementary Table 5), suggesting that nodes in the monsoon-enriched subnetwork are more densely connected between each other than the intermonsoon-enriched subnetwork.

### Different Roles of Rare Taxa and Abundant Taxa in Assembly Processes and the Co-occurrence Network

The null model revealed that CRT experienced more stress of variable selection (63.4%) compared with ART (53.7%) and AT (42.5%) in the total community. The proportion of variable selection in total assembly processes also shared a consistent trend (CRT > ART > AT) in the monsoon community and the intermonsoon community. AT were mainly governed by stochastic processes, including homogenizing dispersal (28.0–46.0%) or weak ecological processes (29.5–69.3%) in the total community, monsoon community, and the intermonsoon community. In addition, rare taxa were more deeply influenced by environmental effects than by spatial effects based on VPA in the total community.

**FIGURE 4:**
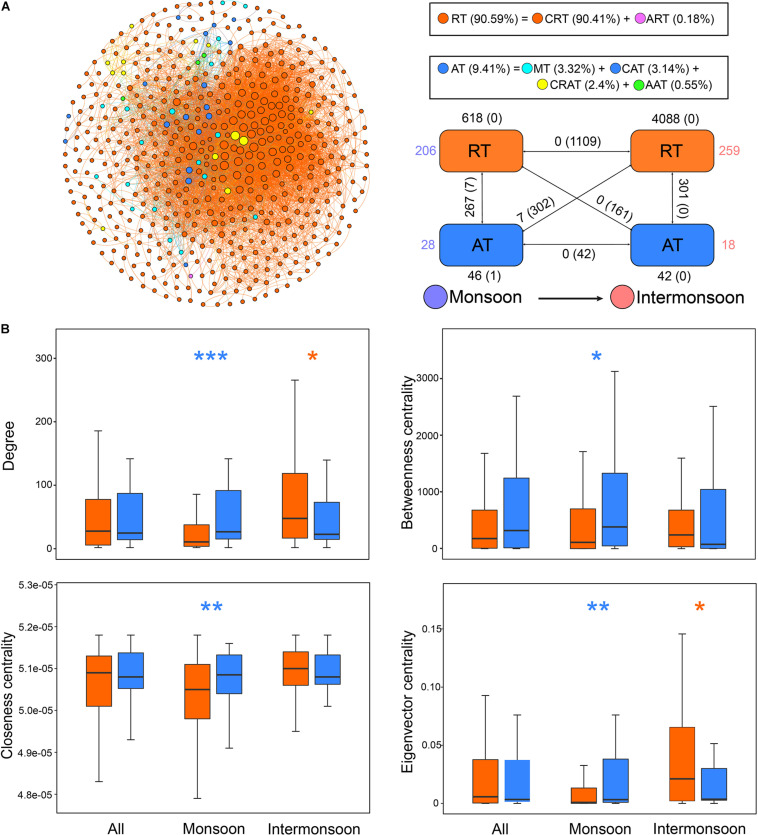
Different roles of rare taxa and abundant taxa in the co-occurrence network. **(A)** The network was conducted by strong (Spearman’s | *r*| > 0.8) and significant (*p*-value < 0.01) correlations between OTUs in the metacommunity. Each node represents an OTU, and each edge represents a correlation. The size of nodes is proportional to the degree of corresponding OTUs (the number of connections). The networks were colored based on the relative abundance of OTUs, showing seasonal variation through the intra-associations within each taxon and inter-associations between two taxa. The rectangle located at the bottom right of the network figure is a summary of the intra-associations and inter-associations. Colored numbers indicate the number of nodes belonging to the corresponding category (i.e., there were 206 RT nodes in the “monsoon” category). Numbers outside and inside parentheses, respectively, indicate the numbers of positive edges and negative edges. Black numbers near the apex of the rectangle in turn indicate the number of intra-associations. Numbers near rectangle edge in turn indicate the number of cross-group interactions. **(B)** Comparison of node-level topological features among metacommunity and subcommunities for rare taxa and abundant taxa. Blue asterisks indicate the values significantly higher in abundant taxa (*p < 0.05; **p < 0.01; ***p < 0.001; Wilcoxon rank-sum test or Brown Mood median test); orange asterisks indicate the properties significantly higher in rare taxa. RT, rare taxa; CRT, conditionally rare taxa; ART, always rare taxa. AT, abundant taxa; MT, moderate taxa; CAT, conditionally abundant taxa; CRAT, conditionally rare and abundant taxa; AAT, always abundant taxa.

Despite their low abundance, a large number of OTUs belonging to CRT were the major nodes in the network, surrounded with several nodes of AT and only one node of ART. Significant difference of the node-level topological features was not observed between RT and AT (Figure 4B), but the AT subnetwork showed slightly higher connectance, clustering coefficient, and modularity than the RT subnetwork (Supplementary Table 5). The mean relative abundance of the intermonsoon-enriched OTUs was significantly lower than that of the monsoon-enriched OTUs (Figure 3A), arising from higher proportion of rare species in the intermonsoon subnetwork (Figure 4A). The value of node-level topological features of RT in the intermonsoon was significantly higher than that in the monsoon, although these features of AT did not show significant seasonal variation (Figure 3B). Moreover, AT occupied a more central position than RT in the monsoon network, while RT seemed to be more important than AT in the intermonsoon network (Figure 4B).

### Drivers Influencing the Seasonal Heterogeneity of the Microbial Community

**FIGURE 5:**
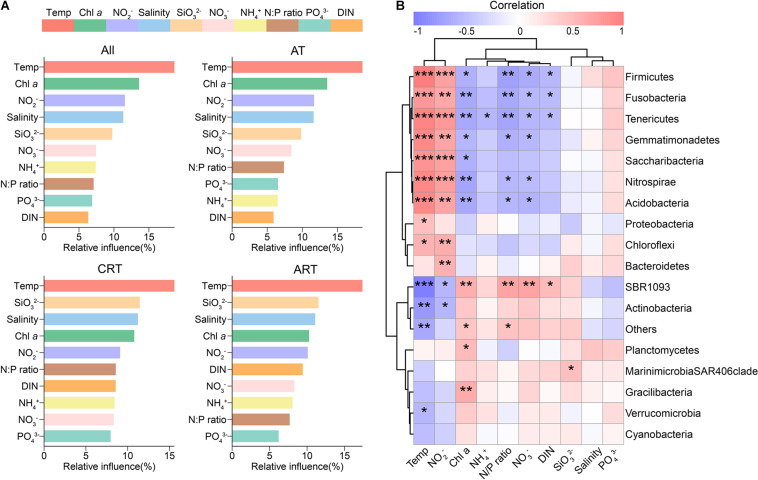
Drivers shaping the microbial communities. **(A)** Aggregated boosted tree (ABT) showing the relative contribution of environmental factors on the community composition. **(B)** The relationships between sea water properties and the relative abundances of microbial phyla based on Spearman correlation (*p < 0.05; **p < 0.01; ***p < 0.001).

Given the predominant deterministic process in the total community, we explored the environmental factors driving community variation. In the present study, sea surface temperature was identified as the most significant factor influencing the seasonal dynamics of the bacterial community (Figure 5 and Supplementary Tables 6, 7). We found a significant phylogenetic signal in OTU temperature niches (Supplementary Figure 7), indicating that closely related species within the bacterial community had more similar responses to temperature variation. In support of our seasonal variation hypothesis, Mantel test exhibited strong and significant correlation between temperature and microbial community structure (Mantel *r* = 0.524; *p* < 0.001), and the correlation in CRT became more significant (Mantel *r* = 0.603; *p* < 0.001) (Supplementary Table 6). Furthermore, temperature can also mediate the balance between determinism/stochasticity in community assembly, resulting in different ecological processes governing RT and AT (Supplementary Figure 10).

Other factors, such as nutrients and Chl *a* concentration, were also identified as significant factors structuring bacterial community. For instance, PO_4_^3–^ and NO_2_^–^ were important predictors for differences in relative abundances of most phyla with a strong and significant linear least-squares regression (Supplementary Figure 11). Differences in PO_4_^3–^ concentrations are positively correlated with the differences in relative abundances of Cyanobacteria, Actinobacteria, Firmicutes, Chloroflexi, etc. In addition, the relative abundances of Proteobacteria showed a positive correlation with ΔNO_2_^–^. The relative abundances of Cyanobacteria were negatively correlated with ΔNH_4_^+^ but positively correlated with ΔChl *a* concentration.

## Discussion

**FIGURE 6:**
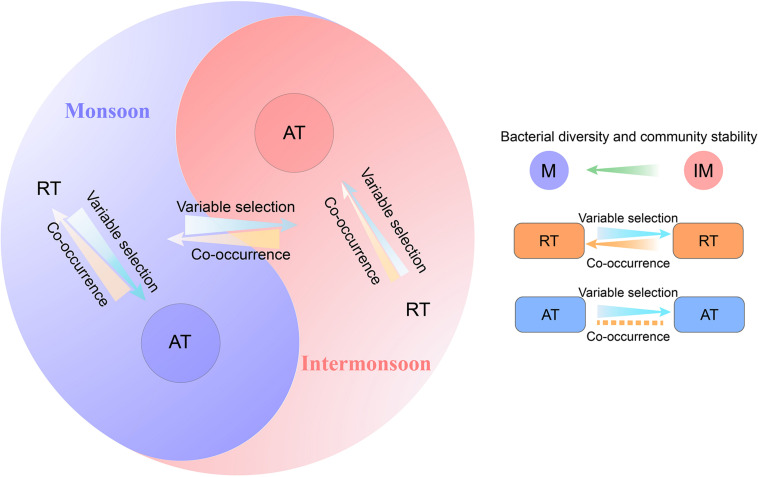
A conceptual paradigm showing the linkage between assembly process and co-occurrence pattern of rare and abundant bacterial community.

Linking community assembly with species co-occurrence can shed light into the underlying mechanisms underpinning the microbial community diversity (Kraft et al., 2008; Valyi et al., 2016; Zhou and Ning, 2017; Jiao et al., 2020). By constructing a link between assembly process and species co-occurrence in the microbial community (Figure 6), we found that (I) deterministic processes (mainly variable selection) became a less dominant assembly process in the intermonsoon communities as microbial co-occurrence increased; (II) this negative tendency between deterministic assembly process and species co-occurrence was observed in rare species rather than abundant species.

### Distinct Assembly Process and Co-occurrence Pattern of the Microbial Community

Quantifying the relative importance of deterministic and stochastic processes is essential in microbial ecology (Kraft et al., 2008; Valyi et al., 2016; Zhou and Ning, 2017; Jiao et al., 2020). Our assessment of assembly processes of bacterial community indicated that the balance between ecological determinism and stochasticity varied with seasons in the EIO. Furthermore, combined with network analysis, we suggested that microbial coexistence is more widespread under weaker environmental filtering. Several studies attempted to unveil the assembly and coexistence mechanisms in microbial ecology (Liu et al., 2020; Zhu et al., 2020; Li et al., 2021), and a similar trend resembling the present study was also reported in agricultural soil ecosystems (Jiao et al., 2020).

The dominant contribution of variable selection in the total community suggested that heterogenous abiotic and biotic factors can impose strong selective pressure by filtering species in the local “seed bank.” Several abiotic factors, such as temperature and nutrients, were identified to be significantly related to the seasonal variability of microbial community structure. These factors have been demonstrated to drive a highly deterministic process in various ecosystems (Pomeroy and Wiebe, 2001; Guo et al., 2014; Xue et al., 2018; Zakem et al., 2018; Jiao and Lu, 2020a; He et al., 2021; Mo et al., 2021; Wan et al., 2021a,b), influencing the diversity of microbial communities (Roberto et al., 2018). For instance, the increase of temperature could cause higher metabolic rates, more rapid succession (Liang et al., 2015; Jiao et al., 2020), and hence more possibilities of species interactions. The bacteria displaying highly specialized thermal niches could result in distinct community composition (Yung et al., 2015), and the species sharing similar thermal preference more likely coexisted in a local community. In addition, in the EIO, the decrease of nutrient concentrations in the surface water, arising from the stratification and increasing stability of upper water from monsoon to intermonsoon, may lead to different environmental stress among microorganisms. For example, heterotrophic bacteria, such as Alpha-, Beta-, and Gamma-proteobacteria, dominated the diazotrophic bacterial community in the EIO during the N-depleted intermonsoon (Wu et al., 2019), owing to their nitrogen fixation ability that can reduce atmospheric N_2_ gas to bioavailable ammonium (Shiozaki et al., 2014).

On the other hand, biotic interactions could influence the microbial community, thus controlling the species co-occurrence simultaneously. It is hard to directly infer the influence of biotic interactions. The significant difference in Chl *a* concentrations between the two seasons might be the consequence of biotic interactions, and correspondingly, changing biomass could also structure distinct bacterial communities. The co-occurrence network provided a statistical inference that both the monsoon and intermonsoon subnetworks showed more positive interactions, indicating the presence of interspecies cooperation and/or similar niche preference (Ju et al., 2014). Multispecies cooperation has been reported to contribute to the resilience and stability of the microbial community (Xue et al., 2018). Moreover, negative correlations accounted for the majority of network edges between seasons, implying the presence of competition and/or seasonal heterogeneity. Interestingly, the highly modularized monsoon subnetwork with low community diversity and connectivity but high nutrients and Chl *a* concentrations was consistent with a laboratory simulation experiment, which demonstrated that high nutrient concentration caused more negative and stronger interactions between species, excluded more species from the community, resulted in a loss of biodiversity, and decreased the stability of the microbial communities (Ratzke et al., 2020), while the oligotrophic conditions may enhance the cooperative interactions (e.g., cross-feeding) in the intermonsoon communities. Besides, it is likely that other biotic interactions, such as top-down control (e.g., predation and bacteriophage lysis), may also contribute to the community assembly and species co-occurrence (Berga et al., 2015).

Stochastic processes could be the dominant assembly process in some cases, and they might yield complex microbial diversity (Matthews and Whittaker, 2014; Zhou and Ning, 2017) and co-occurrence patterns similar to the results driven by deterministic processes. In this study, homogenizing dispersal rather than dispersal limitation was identified as an important assembly process structuring the community diversity. Homogenizing dispersal results from high levels of organism exchange (Stegen et al., 2015). Ocean current transportation and frequently occurred mesoscale surface ocean features (eddies) in the EIO (Jyothibabu et al., 2017) may introduce more exchanges of water mass and change the opportunities of species interactions. Several research have proved the importance of stochastic processes. For example, Shi et al. (2018) reported that stochastic processes would play a stronger role with little environmental differences and selection pressure. Accompanied by the minor roles of exogenous factors in shaping the communities, Chen et al. (2019) found that microeukaryotic communities were strongly driven by stochastic processes, explaining nearly 90% of the community variation by the neutral community model during wet and dry seasons in the Tingjiang River. In addition, Roguet et al. (2015) demonstrated that the composition and structure of bacterial community was mainly governed (76%) by stochastic processes in 49 lakes of Paris area, France. In fact, compared with macroorganisms, microorganisms possess not only a higher degree of physiological tolerance and metabolic flexibility but also high dispersal rates, rapid growth rates, and, importantly, evolutionary adaptation obtained from horizontal gene transfer (Allison and Martiny, 2008; Fuhrman et al., 2015). These features enable them to occupy wider niches, generating high diversity and coexistence of microbial communities (Leibold and McPeek, 2006; Stegen et al., 2012; Lange et al., 2015).

### Linking Community Assembly and Species Co-occurrence for Rare and Abundant Microbial Community

Illustrating the mechanisms underpinning the diversity of microbial communities, especially for the rare and abundant components, is crucial to comprehensively understand the marine ecosystem. Therefore, we employed the link between community assembly and species co-occurrence considering the roles of rare and abundant components separately.

As expected, CRT, representing the rare species that might be favored occasionally (Caron and Countway, 2009; Konopka et al., 2015), were more easily controlled by deterministic processes of community assembly (mainly variable selection in this study) in response to changing environments. Li et al. (2021) applied a phylogenetic null model and network analysis to examine the direct and/or indirect cooperation between methanogenic archaeal taxa in rice paddies across the Asian continent, and they demonstrated that the relative importance of determinism and stochasticity differed between commonly coexisted taxa and endemically coexisted taxa. Similarly, Mo et al. (2020) investigated the biogeography and co-occurrence patterns of marine bacteria from three subtropical bays, and they found that deterministic processes played a greater role in the community variation of specialists (84%) compared with generalists (56%). Indeed, rare species were thought to have less competitive capability to utilize resources compared with abundant species, as well as intrinsically low growth rates (Logares et al., 2015). Despite their low abundance, rare species can exert a disproportionate effect on the functional stability of the microbial community (Konopka et al., 2015) and significantly contribute to the temporal changes in the microbial community (Shade et al., 2014; Xue et al., 2018). On the contrary, AT are usually defined as environmental generalist, having wider ecological niches with absolute numerical advantage (Mo et al., 2020). They have also been reported to possess stronger environmental adaptation than rare species in agricultural soils across eastern China and wetland soils from the Qinghai–Tibet Plateau (Jiao and Lu, 2020b; Wan et al., 2021b).

On the other hand, we found that the co-occurrence pattern of RT and AT was not constant under different environmental conditions. Specifically, the centralities of AT were almost similar between the two seasons, while the relative centralities of RT varied in different seasonal networks. It has been recently reported that the co-occurrence pattern of bacterial generalists was different from specialists in complexity and stability, and specialists possessed a more complex co-occurrence pattern than generalists (Mo et al., 2020). The coexistence of the microbial community is regarded as a result of the balance between niche differences and fitness differences (HilleRisLambers et al., 2012). Thus, the changing centralities of RT/AT in the co-occurrence network might arise from the difference of environmental filtering between the two seasons. Also, it was notable that neutral processes can act concurrently to modulate the occurrence frequency of species due to random fluctuations of the microbial community (Chen et al., 2017). In this case, rare species will disappear due to ecological drift, while abundant species are easy to disperse by chance and occur in more sites (Chave, 2004; Sloan et al., 2006; Bahram et al., 2016).

These results and assumptions together with previous paradigms (Jiao et al., 2020) lead us to propose a modified conceptual framework (Figure 6). By incorporating rare and AT into the linkages between assembly processes and species coexistence, the present study could improve our understanding of microbial communities in marine ecosystems for better response to climate change. Although the framework offers a potentially novel insight, we acknowledge several limitations in the study. For example, the framework is inferred based on limited data sets in the monsoon and intermonsoon. Exogenous selective forces may shift in strength and duration, with periodicities on differing time scales (Bell, 2010) and heterogeneities on differing space scales. The ecological patterns of different taxa may vary with the changing environment spanning different spatiotemporal scales. Meanwhile, samples from a single depth without multiple layers may make the results hard to represent the upper Indian Ocean (e.g., whether this framework suiting the DCM layer needs further research). Therefore, more samples based on large spatiotemporal scale are needed to comprehensively depict the horizontal, vertical, and seasonal variation of the microbial community in the EIO. Moreover, the assembly and co-occurrence patterns of abundant and rare bacterial community under different environments described here need to be validated with more research across a broad range of ecosystems.

## Data Availability Statement

The raw data have been submitted to NCBI Sequence Read Archive (SRA) under Bioproject accession PRJNA607183.

## Author Contributions

LL, CW, and JS conceived and designed the experiments. LL wrote the manuscript. CW, WX, and HL carried out the sample collection. LP extracted DNA. CG, GZ, CW, WX, HL, and XW determined the environmental parameters. LL and DH analyzed the data. LL, JS, MW, LP, CW, and YW contributed to manuscript development and revisions. All authors read and approved the final manuscript.

## Conflict of Interest

The authors declare that the research was conducted in the absence of any commercial or financial relationships that could be construed as a potential conflict of interest.

## Publisher’s Note

All claims expressed in this article are solely those of the authors and do not necessarily represent those of their affiliated organizations, or those of the publisher, the editors and the reviewers. Any product that may be evaluated in this article, or claim that may be made by its manufacturer, is not guaranteed or endorsed by the publisher.

## Footnotes

1
http://www.r-project.org/
