# Supplementary material for: Assembly Processes and Co-occurrence Patterns of Abundant and Rare Bacterial Community in the Eastern Indian Ocean
Source: Front Microbiol. 2021 Aug 11;12:616956. doi: 10.3389/fmicb.2021.616956 (PMC8385211; doi:10.3389/fmicb.2021.616956)
Supplement: Supplementary file 1 [file Data_Sheet_1.doc]

**Journal: *Frontiers in Microbiology***

*Supporting information of the article:*

**Assembly processes and co-occurrence patterns of abundant and rare bacterial community in the eastern Indian Ocean**

Liuyang Li^1,2^, Laxman Pujari^1^, Chao Wu^1^, Danyue Huang^1,2^, Yuqiu Wei^1,3^, Congcong Guo^1,3^, Guicheng Zhang^1^, Wenzhe Xu^1^, Haijiao Liu^1^, Xingzhou Wang^1,4^, Min Wang^5^, Jun Sun^1,6*^

**^1^** *Research Centre for Indian Ocean Ecosystem, Tianjin University of Science and Technology, Tianjin 300457, China*

**^2^** *School of Life Sciences & Biotechnology, Shanghai Jiao Tong University, Shanghai, PR China*

**^3^***Institute of Marine Science and Technology, Shandong University, 72 Binhai Road, Qingdao 266200, China*

**^4^** *College of Biotechnology, Tianjin University of Science and Technology, Tianjin 300457, China*

**^5^** *College of Marine Life Sciences, Institute of Evolution and Marine Biodiversity, Ocean University of China, Qingdao 266003, China*

**^6^** *College of Marine Science and Technology, China University of Geosciences (Wuhan), Wuhan, Hubei 430074, China*

**Running title:** Assembly and coexistence of marine bacteria

* Corresponding author. Tel.: +86 222 60601101;

E-mail address: phytoplankton@163.com (J. Sun).

**This supplementary information contains:**

- 25 Pages
- 13 Figures
- 7 Tables
- 8 references

**Explanation for sampling deviation and statistical verification**

Overall, this study showed that the influence of seasonal dynamics was much stronger than spatial heterogenity on bacterial communities. Given that the spatial distribution of the samples from the two seasons was not strictly matched (Supplementary Fig. S1), we carried out rigorous statistical verification to confirm that the diversity of bacterial communities were undoubtedly controlled by seasonal variation, and only little influence was introduced by spatial deviation.

In detail, we performed two-way analysis of variance (two-way ANOVA), non-metric multidimensional scaling (NMDS), hierarchical clustering analysis (HCA), nonparametric analysis of similarity (ANOSIM), and variation partitioning analysis (VPA) to validate seasonal effect rather than spatial deviation controlled the diversity of bacterial communities. Alpha-diversity indices were calculated at OTU level by using diversity function in “vegan” package (Oksan*en et a*l., 2010). Spatiotemporal effects on alpha-diversity were examined by two-way ANOVA. The data that failed for the testing of normality (Shapiro-Wilk test) and homogeneity of variance (Bartlett test) were further tested using nonparametric Scheirer-Ray-Hare test (Sokal, 1995) and Kruskal-Wallis test (Fie*ld et a*l., 2012). To further interpret the spatiotemporal distribution patterns of the bacterial communities and the similarity of samples to each other, NMDS and HCA were used to explore bacterial community characters based on Bray–Curtis dissimilarities. Unweighted pair-group method with arithmetic means (UPGMA) was analyzed using hclust function in “vegan” package in R. Differences in bacterial communities between spatiotemporal groups were investigated using nonparametric ANOSIM (permutations = 999) by the anosim function in “vegan” package (Clarke, 1993). Finally, to distinguish the relative contribution of seasonal and spatial effects on community diversity, VPA was applied by separating community variation into seasonal effect, spatial effect and environmental effect. Independent spatial variation that excludes the seasonal component represents the spatial effect. Similarly, independent seasonal variation without a spatial component corresponds to the seasonal effect.

The result of ANOVA showed that alpha-diversity indices were significantly correlated with season rather than latitude (Supplementary Table S2), implying a strong seasonal effect dominating community variation rather than spatial deviation during sampling. We further used NMDS analysis combined with ANOSIM and UMGMA analysis to confirm that seasonal effects rather than spatial effects exerted a greater influence on bacterial community structure (Supplementary Table S3, Fig. S5, and Fig. S12). The NMDS analysis showed a strong seasonal separation pattern for CRT, ART and AT (Supplementary Fig. S5). ANOSIM analysis supported the seasonal dynamics in microbial community that bacterial communities were mainly governed by temporal factors according to the between-group distances (Supplementary Table S3). Importantly, according to VPA (Supplementary Fig. S13 and Table S4), the seasonal effect was 5.75 times greater than the spatial effect, suggesting that spatial variation only had little influence on the community structure. One may argue that the distinct bacterial community composition could also be shaped by the variation of environmental conditions between different stations. Our VPA result also suggested that season-induced environmental effects were significantly higher than the space-induced environmental effects (15.2% versus 2.2%). It is mentioned that we have not detected dispersal limitation using null model based on phylogenetic turnover (Fig. 2).

Our statistical verification concluded both alpha-diversity, beta-diversity, phylogenetic turnover and environmental factors to confirm whether seasonal variation or spatial deviation drive the community structure. Given the bacterial community in the EIO were highly seasonal structured, we hypotheses that the deviation of sampling sites only exerts little effect on community seasonal dynamics. None or little dispersal limitation detected in the null model further confirmed that the bacterial communities in our study were highly dispersed. And the dispersal process could decrease the influence of sampling deviation. Such a high dispersal process may arise from the easily dispersed medium (in water) and carrier (very small size of bacteria). Furthermore, ocean currents and frequently occurred mesoscale surface ocean features may bring a large number of possibilities for bacterial immigration, and promote the cross-regional distribution of microbial community. For example, previous study suggested that diazotrophs can remain active after being transported over great distances by ocean currents (Shioza*ki et a*l., 2013).


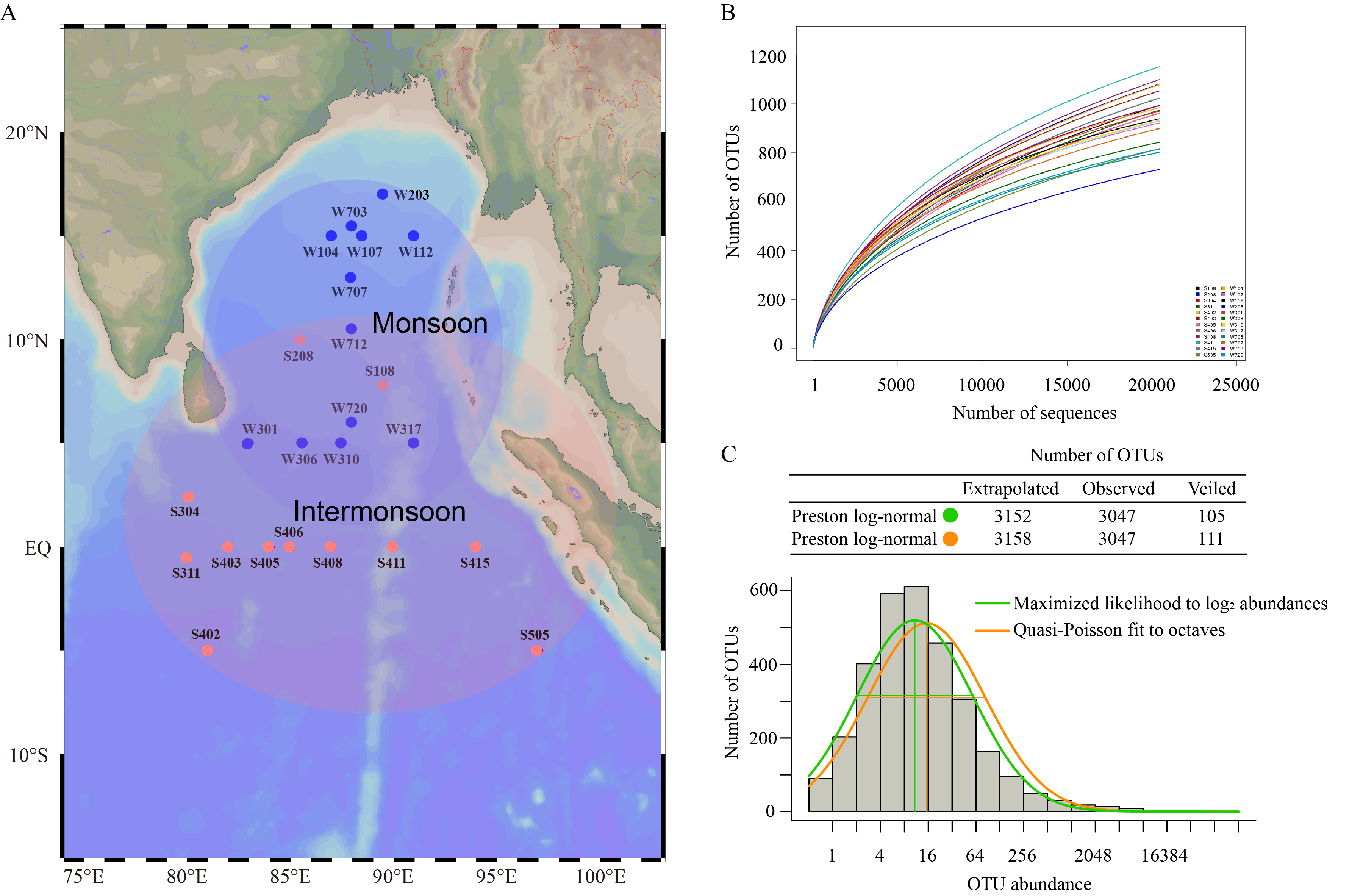


**Fig. S1** Bacterial diversity in the eastern India Ocean (EIO). **A** The sampling seasons and locations in the EIO. **B** Rarefaction curves for each station of operational taxonomic unit (OTU) at 97% sequence similarity level. **C** The distribution of OTU abundance and fit to the Preston log-normal model. The green line and orange line represent two methods: maximized likelihood to log_2_ abundances and Quasi-Poisson fit to octaves, respectively. The values of Preston veil (Extrapolated OTUs minus Observed OTUs) reflect the OTUs number that we did not capture during our sampling. The low Preston veil values indicate the existence of adequate bacterial richness, supporting our extraction of general patterns of bacterial biodiversity from dataset


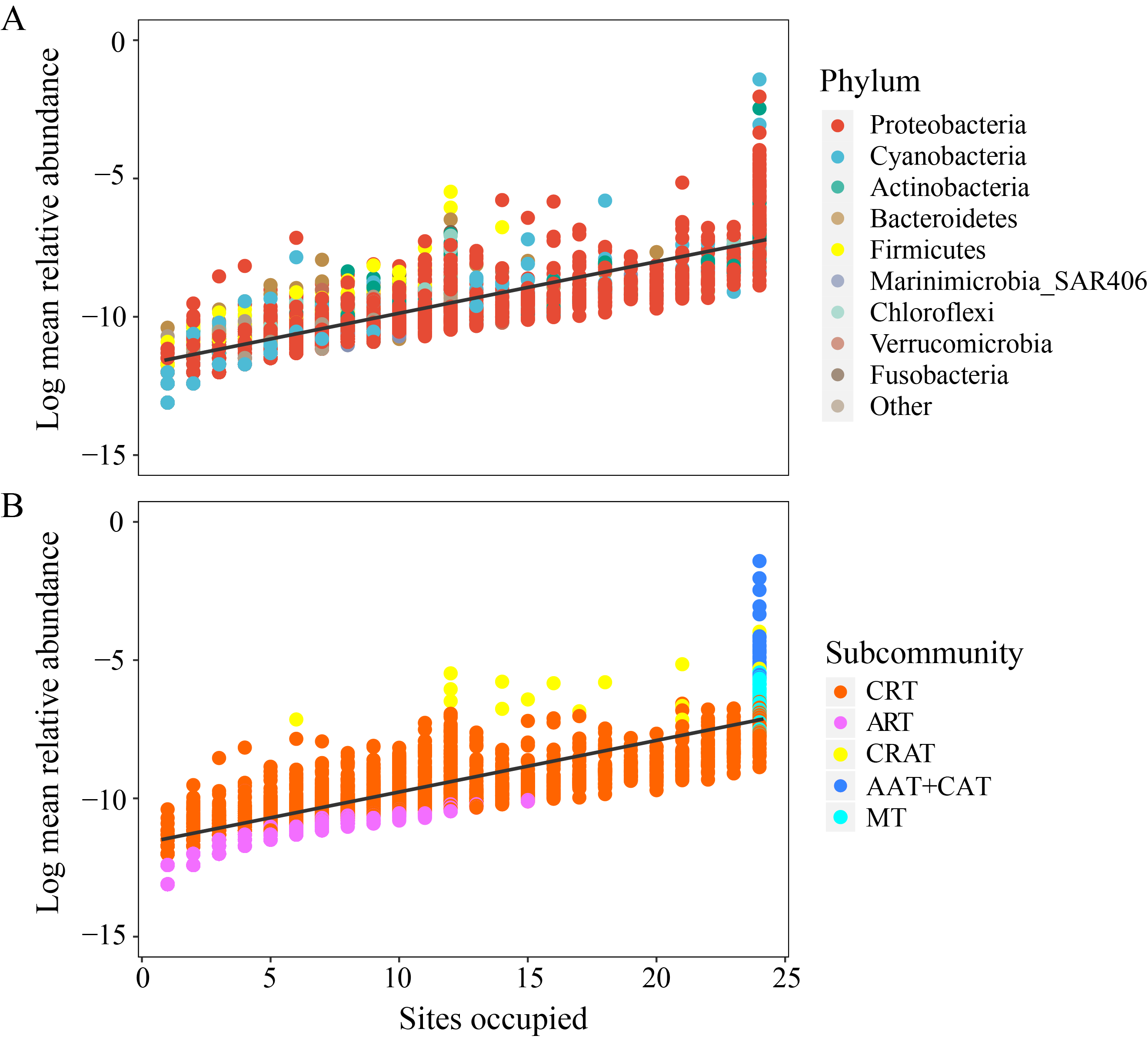


**Fig. S2** Spearman’s rank correlation between the relative abundance of OTUs and the number of sites occupied. The figures are colored based on the bacterial taxonomic composition of each OTU and their relative abundance, respectively. CRT, conditionally rare taxa; ART, always rare taxa. AT, abundant taxa, including AAT (always abundant taxa), CAT (conditionally abundant taxa), CRAT (conditionally rare and abundant taxa) and MT (moderate taxa).


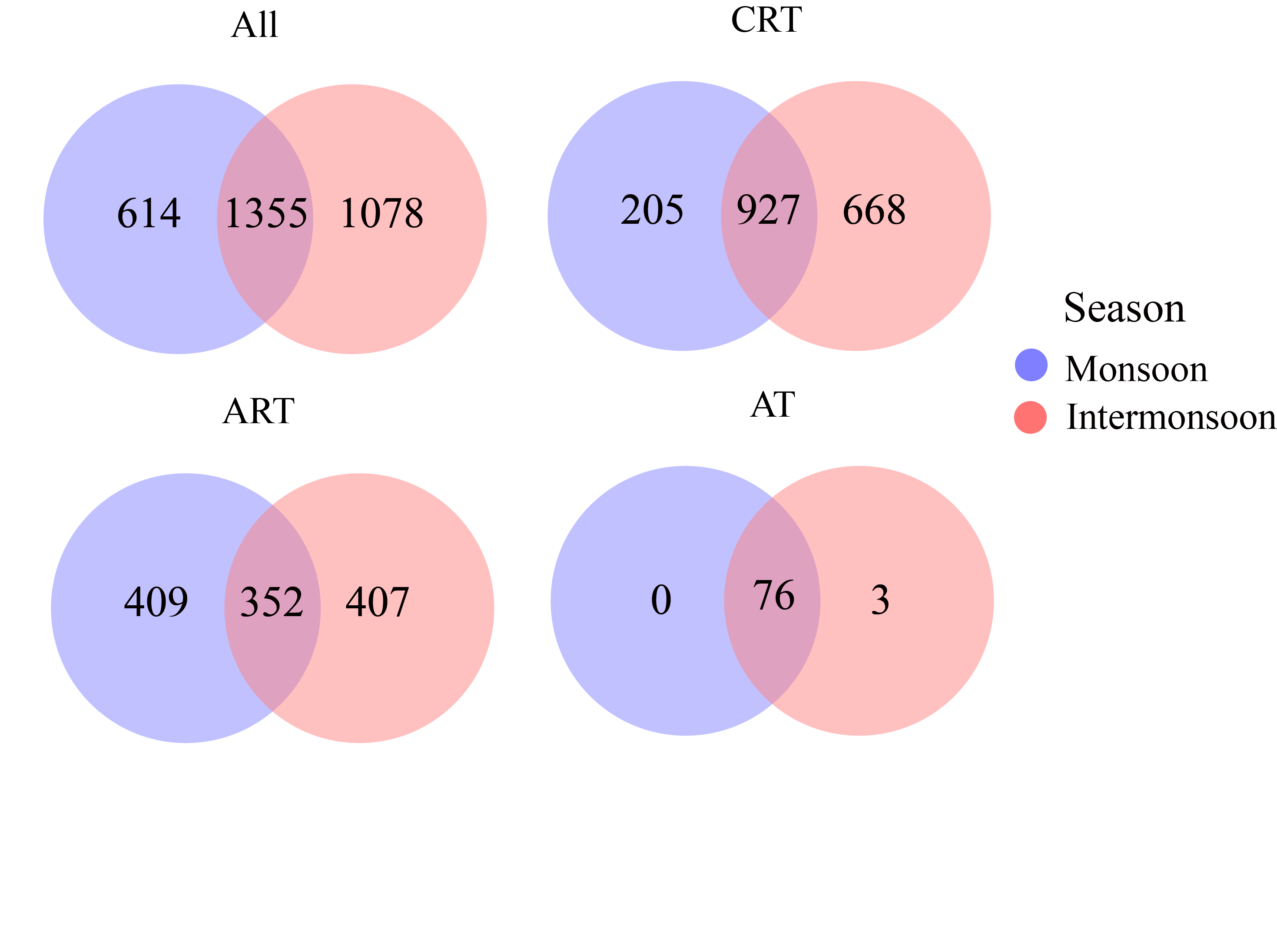


**Fig. S3** Venn diagram presenting the numbers of unique and shared OTUs between two seasons. All-whole bacterial community; CRT-conditionally rare taxa; ART-always rare taxa; AT-abundant taxa


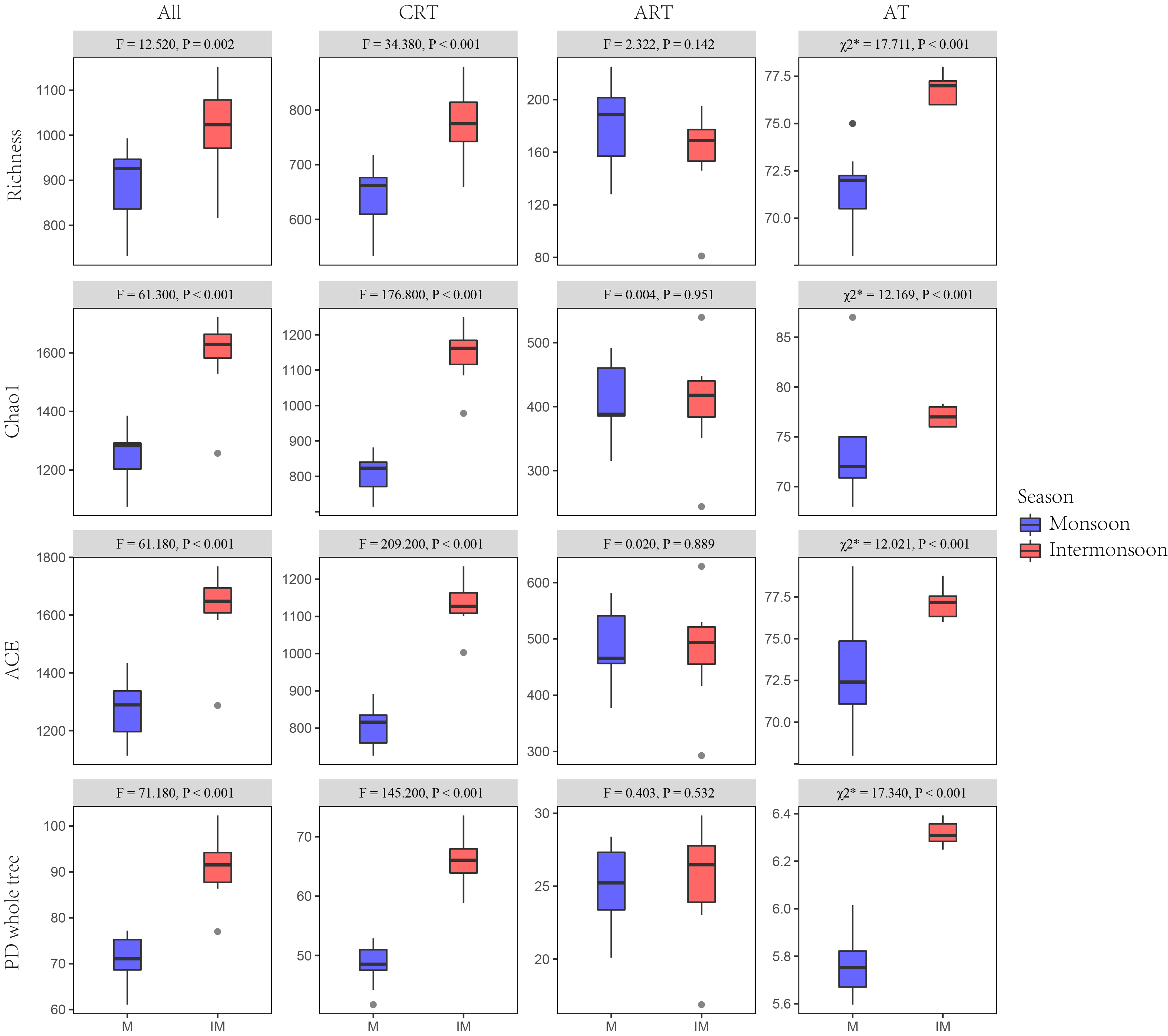


**Fig. S4** Seasonal variation in alpha-diversity. One-way ANOVA was used to compare the differences between different periods. *The data that failed for the test of normality (Shapiro-Wilk test) and homogeneity of variance (Bartlett test) were further tested using nonparametric Kruskal-Wallis test. F: Two-way ANOVA; χ^2^*: Kruskal-Wallis test. All-whole bacterial community; CRT-conditionally rare taxa; ART-always rare taxa; AT-abundant taxa


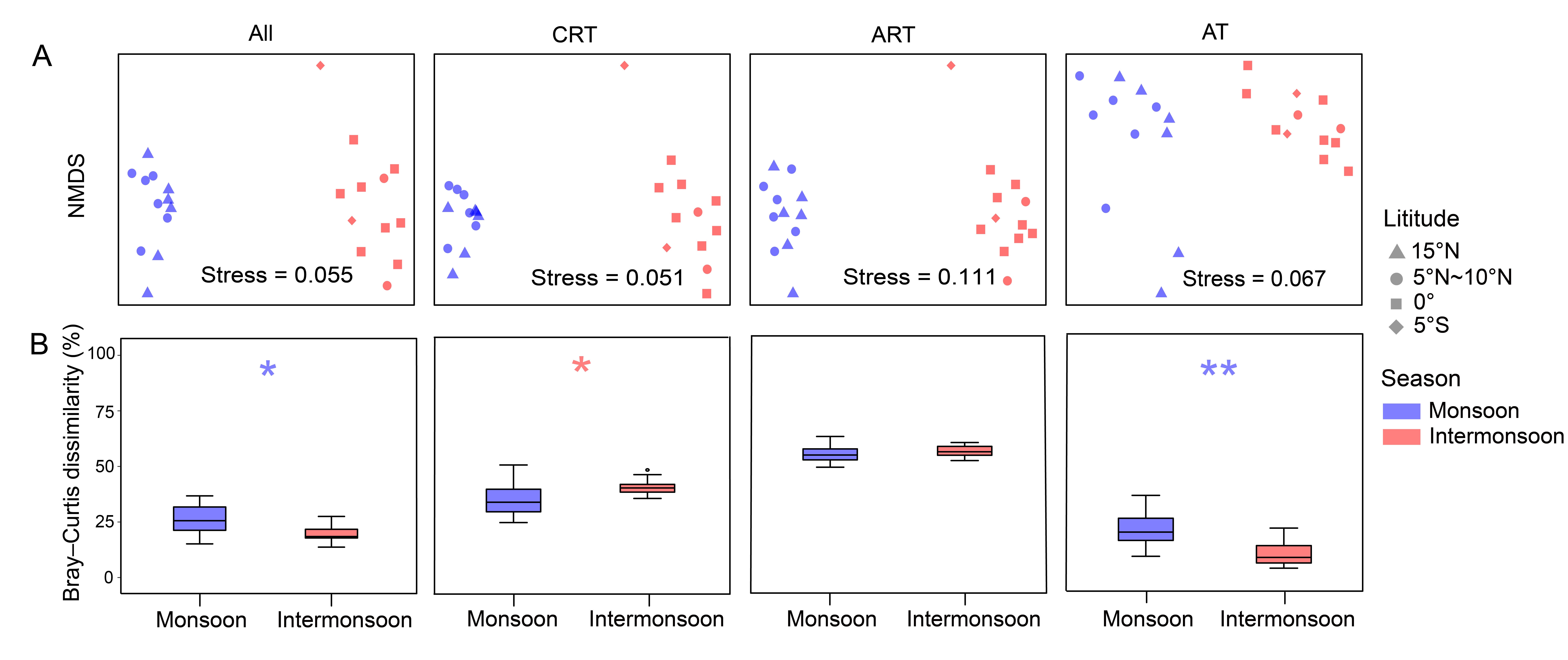


**Fig. S5** The patterns of bacterial community beta-diversity in the monsoon and intermonsoon. **A** Non-metric multidimensional scaling (NMDS) analysis of bacterial communities based on Bray–Curtis dissimilarity. The shape and color of each sample are based on their spatiotemporal characters. Triangle, circle, square and diamond represent the region located near 5°S, 0°, 5°N~10.5°N and 15°N in Eastern India Ocean, respectively. Blue and red represent the monsoon and intermonsoon, respectively. **B** The pairwise Bray–Curtis dissimilarity of rare and abundant bacterial communities between different periods. The lines within each box stand for the median values (n = 12), the top and bottom boundaries of each box represent the upper and lower quartile (75th and 25th) values, respectively. Significant difference was performed according to nonparametric Wilcoxon rank sum test (**P* < 0.05; ***P* < 0.01). All, whole bacterial community; CRT, conditionally rare taxa; ART, always rare taxa; AT, abundant taxa


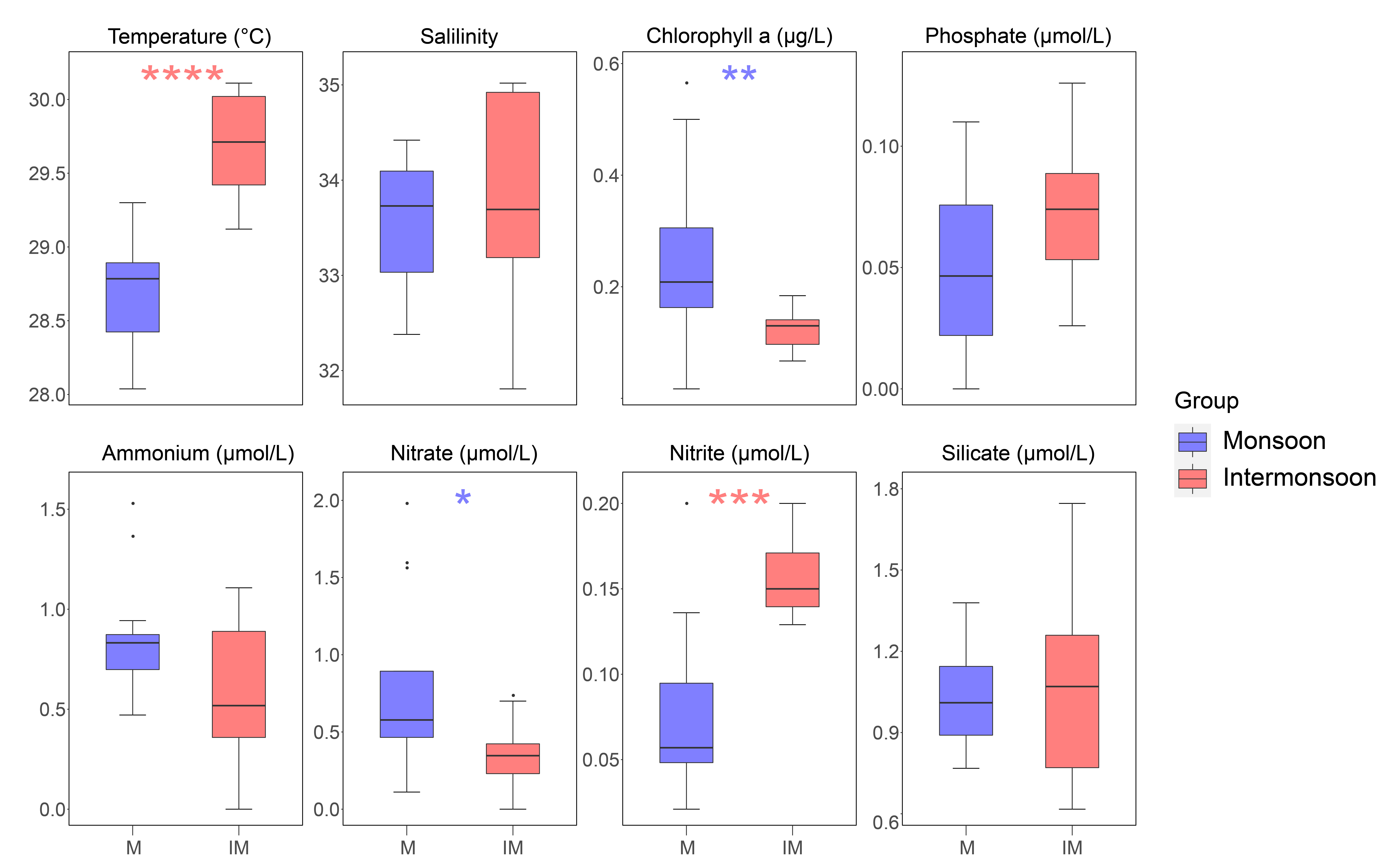


**Fig. S6** The boxplot showing the seasonal variation of environmental parameters in the Eastern India Ocean (EIO). The lines within each box stand for the median values (n = 12), the top and bottom boundaries of each box represent the upper and lower quartile (75th and 25th) values, respectively. Blue asterisks indicate the values significantly higher in the monsoon (*P < 0.05; **P < 0.01; ***P < 0.001; ****P < 0.0001; Wilcoxon rank-sum test); red asterisks indicate the properties significantly higher in the intermonsoon.


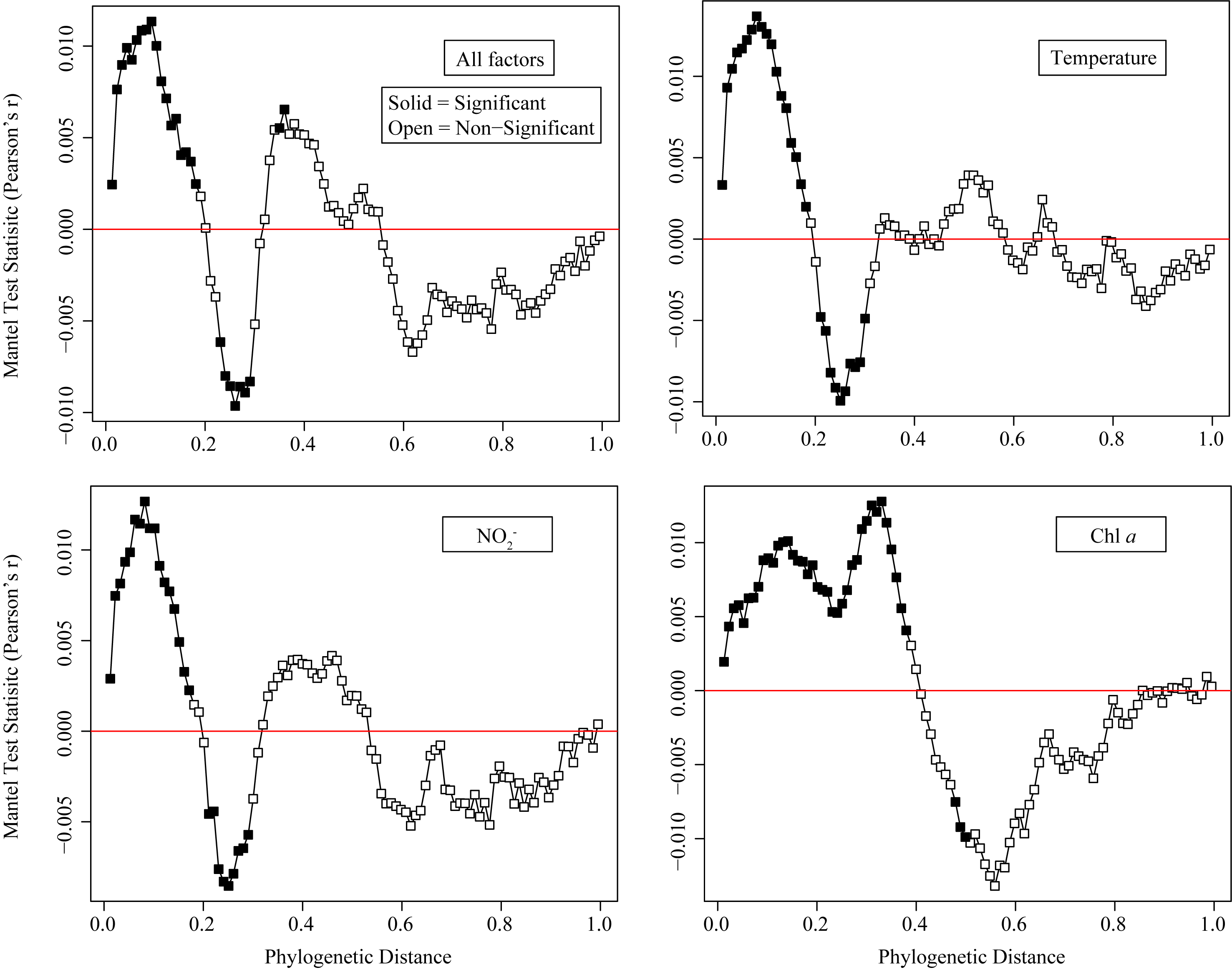


**Fig. S7** Phylogenetic mantel correlogram evaluating significant phylogenetic signal across short phylogenetic distances in the bacterial community. Environmental optima based on all factors and three important factors (temperature, Chl *a* and nitrite) were estimated for each species, respectively. Solid and open symbols represent significant (*P* < 0.05, 999 permutations) and nonsignificant correlations between-OTU niche differences and between-OTU phylogenetic distances, respectively. The phylogenetic distances were partitioned into 100 classes by 0.01 units. Significantly positive correlations indicate that there is phylogenetic signal in OTU environmental niches, but only across short phylogenetic distances


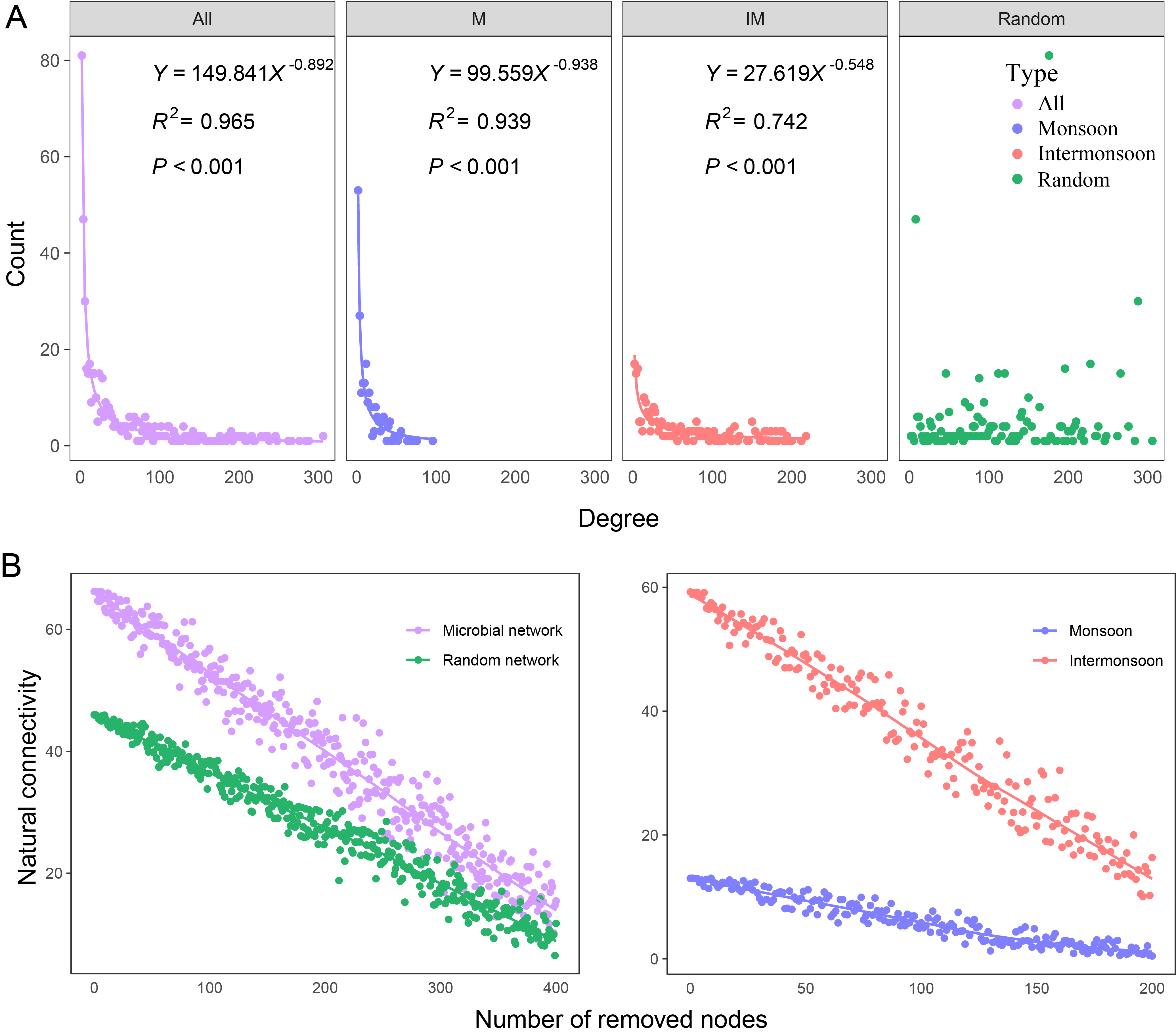


**Fig. S8** The power law fits (**A**) and natural connectivity (**B**) of the real/random networks, and the monsoon/intermonsoon networks


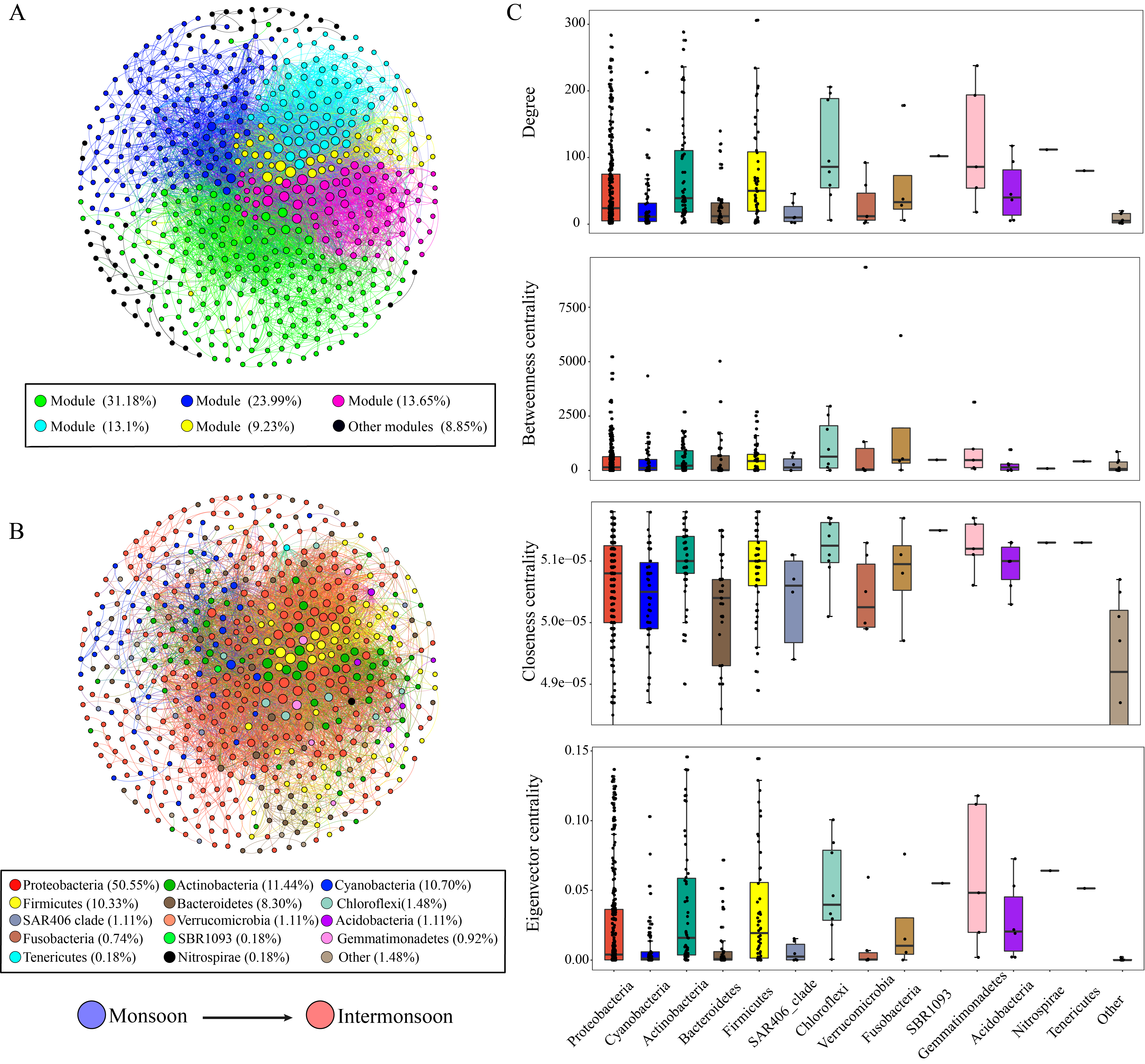


**Fig. S9** The highly modular co-occurrence network and its taxonomic composition. The network was conducted by strong (Spearman’s |r| > 0.8) and significant (P-value < 0.01) correlations between OTUs in the metacommunity. Each node represents an OTU, and each edge represents a correlation. The size of nodes is proportional to the degree of corresponding OTUs (the number of connections). The nodes are colored according to taxonomic composition (**A**) and different types of modularity classes (**B**), respectively. **C** Comparison of node-level topological features among different phyla


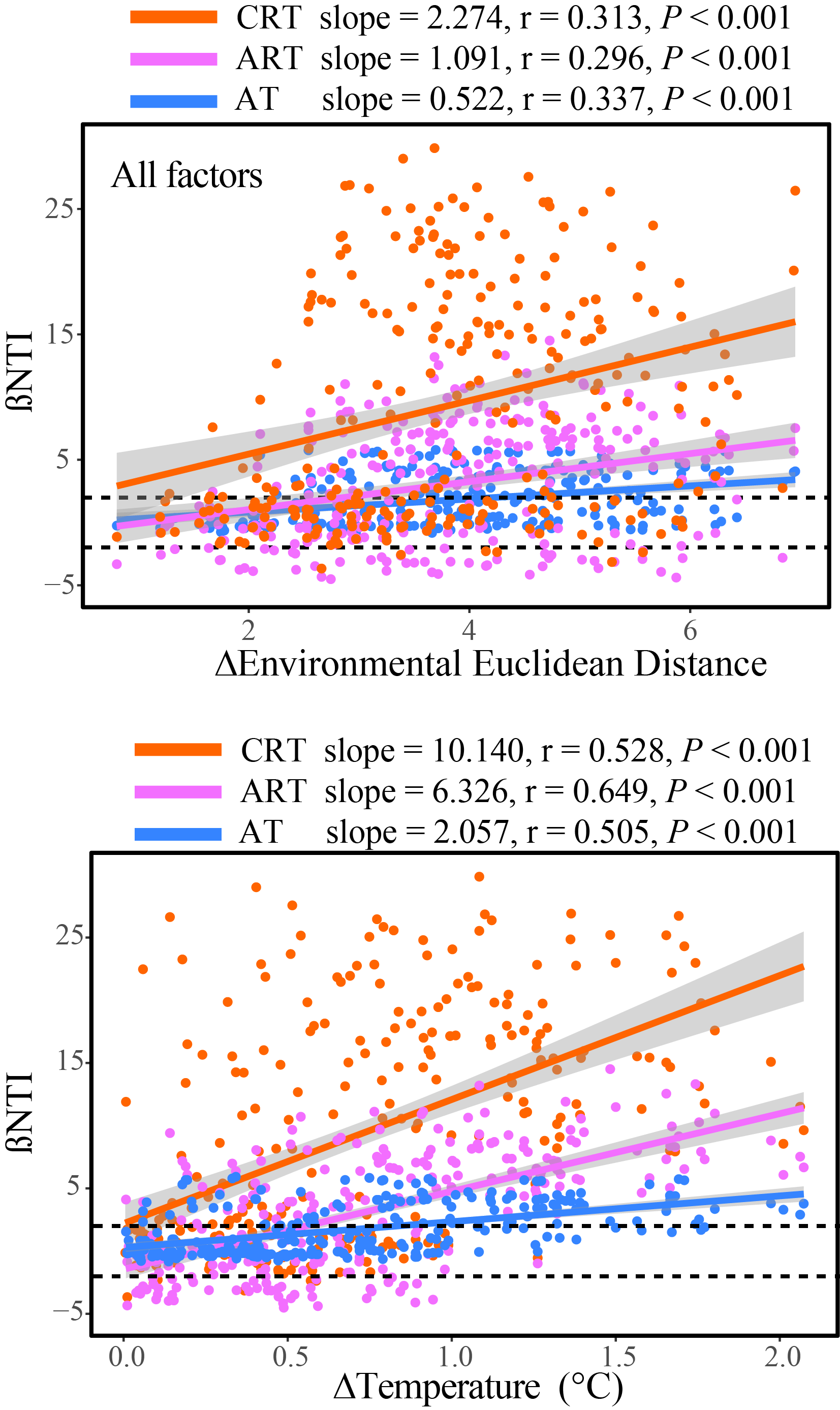


**Fig. S10** Significant difference of determinism for CRT, ART and AT in relation to the differences in environmental factors (temperature). CRT, conditionally rare taxa; ART, always rare taxa; AT, abundant taxa


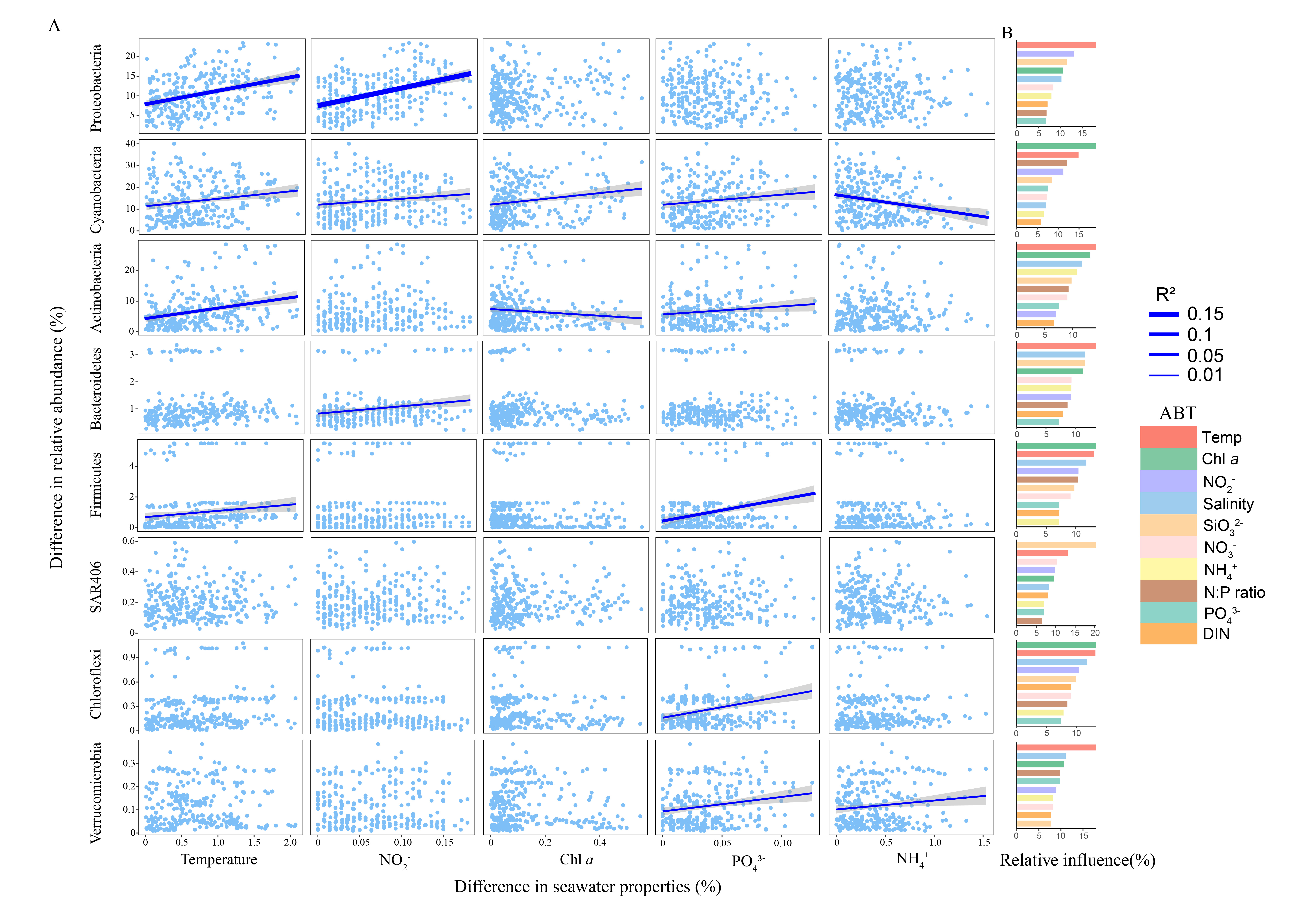


**Fig. S11** Drivers of different bacterial phyla. **A** The linear least-squares regression of the differences in relative abundances of primary bacterial phyla and the differences in seawater properties **B** Aggregated boosted tree (ABT) showing the relative contribution of the environmental factors on the composition of primary bacterial phyla.


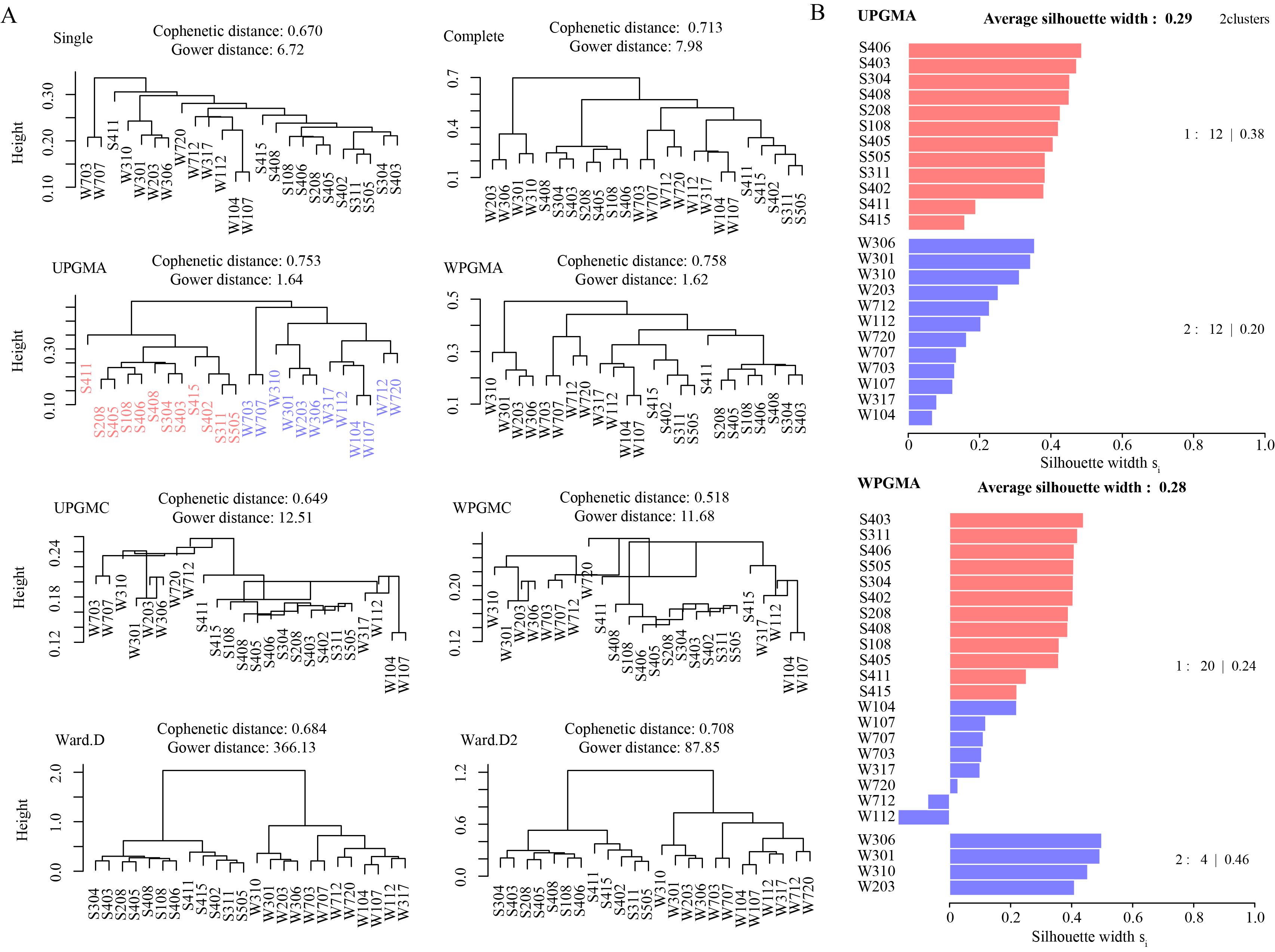


**Fig. S12** **A** The results of several types of hierarchical clustering models at the OUTs level. The clustering methods with the higher Cophenetic values and minor Gower distances are regarded as the optimal clustering models. Single, single linkage agglomerative clustering; complete, complete linkage agglomerative clustering. Four types of average agglomerative clustering methods: UPGMA, unweighted pair group method using arithmetic means; WPGMA, weighted pair group method using arithmetic means; UPGMC, unweighted pair group method of centroid; WPGMC, weighted pair group method using centroid. And 2 kinds of minimum variance clustering approaches, Ward and Ward.D2. **B** The plot showing the silhouette width value of each site according to UPGMA and WPGMA methods (2 clusters). The values of silhouette width reflect the fits to current cluster. Silhouette width less than 0 represents potential misallocation. UPGMA was finally selected as the optimal clustering method according to Cophenetic distance, Gower distance and Silhouette width.

**
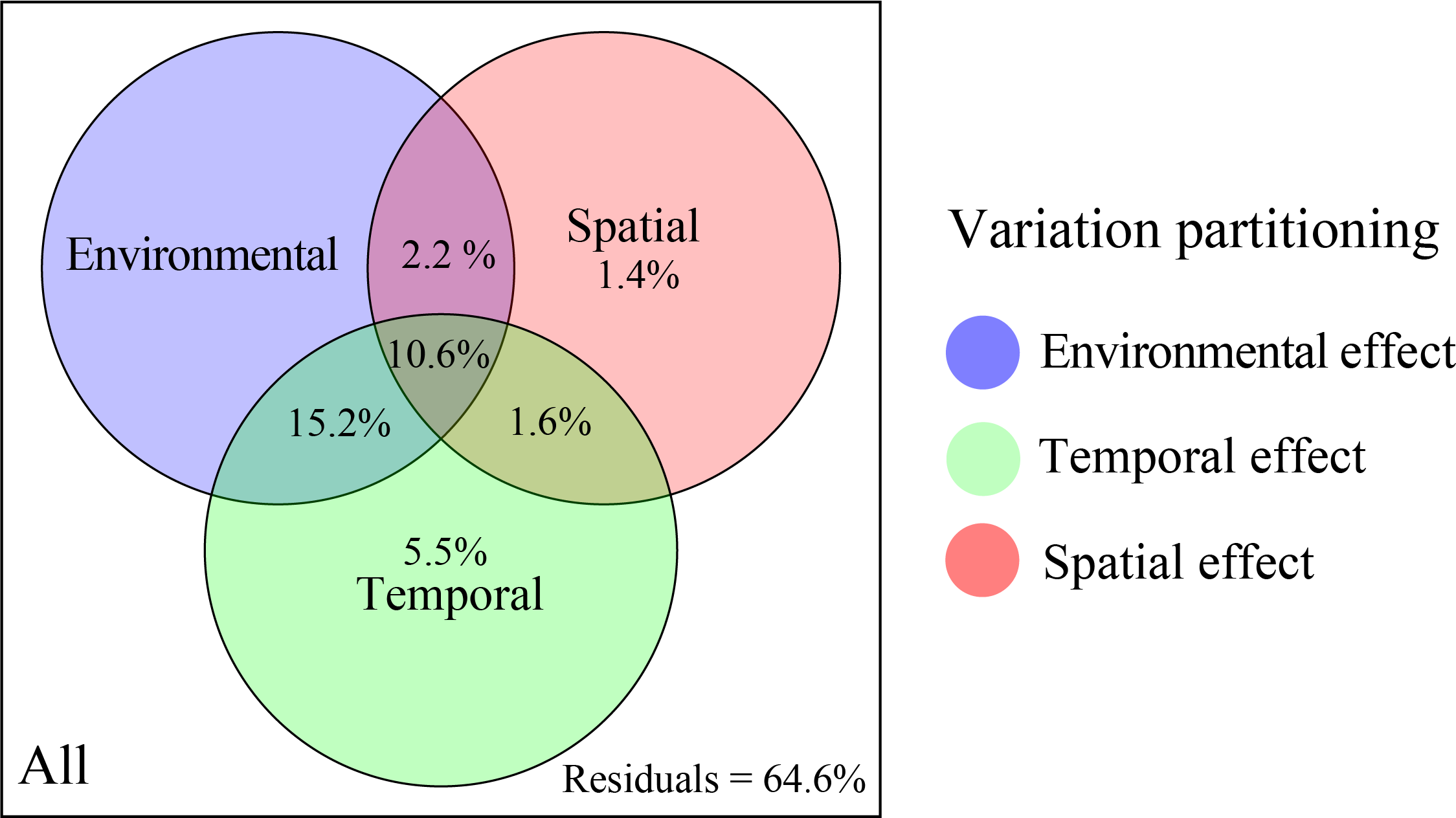
**

**Fig. S13** Variation partitioning analysis (VPA) showing the relative importance of seasonal and spatial effects on community diversity. The higher residuals may come from uncaptured variables and stochastic processes of microbial community

**Table S1** The richness and abundance of different taxa of bacterial OTUs data sets at 97% similarity level

| Category | OTU number | Sequence number |
| --- | --- | --- |
| All | 3047 | 490,704 |
| CRT | 1800 (59.07%) | 81467 (16.60%) |
| ART | 1168 (38.33%) | 5069 (1.03%) |
| AT | 79 (2.59%) | 404168 (82.36%) |

All, whole bacterial community taxa

CRT, conditionally rare taxa

ART, always rare taxa

AT, abundant taxa, including always abundant taxa (AAT), conditionally abundant taxa (CAT), conditionally rare and abundant taxa (CRAT) and moderate taxa (MT)

**Table S2** Two-way ANOVA and Scheirer-Ray-Hare test showing the spatialtemporal effects on the alpha-diversity of bacterial community.

|  | All | |  | | CRT | | |  | | ART | | |  | | AT | | |  |
| --- | --- | --- | --- | --- | --- | --- | --- | --- | --- | --- | --- | --- | --- | --- | --- | --- | --- | --- |
|  | F/H***** | P | |  | | F/H***** | P | |  | | F/H***** | P | |  | | F/H***** | P | |
| Time |  |  | |  | |  |  | |  | |  |  | |  | |  |  | |
| Richness | 12.028 | **0.003** | |  | | 31.900 | **<0.001** | |  | | 2.491 | 0.131 | |  | | 17.711***** | **<0.001** | |
| Shannon | 0.667 | 0.424 | |  | | 1.215 | 0.284 | |  | | 2.702 | 0.117 | |  | | 0.963***** | 0.326 | |
| Simpson | 0.815 | 0.378 | |  | | 0.605 | 0.446 | |  | | 2.803***** | 0.094 | |  | | 2.083***** | 0.149 | |
| Chao1 | 73.896 | **<0.001** | |  | | 179.045 | **<0.001** | |  | | 0.005 | 0.942 | |  | | 12.169***** | **<0.001** | |
| ACE | 61.074 | **<0.001** | |  | | 190.704 | **<0.001** | |  | | 0.027 | 0.871 | |  | | 12.021***** | **<0.001** | |
| Goods_coverage | 92.255 | **<0.001** | |  | | 45.550 | **<0.001** | |  | | 2.120 | 0.162 | |  | | 0.631***** | 0.427 | |
| Pielou | 2.428 | 0.136 | |  | | 6.363 | **0.021** | |  | | 0.120***** | 0.729 | |  | | 2.430***** | 0.119 | |
| PD_whole_tree | 74.280 | **<0.001** | |  | | 145.495 | **<0.001** | |  | | 0.462 | 0.505 | |  | | 17.34***** | **<0.001** | |
| Space |  |  | |  | |  |  | |  | |  |  | |  | |  |  | |
| Richness | 0.715 | 0.555 | |  | | 0.472 | 0.705 | |  | | 1.534 | 0.238 | |  | | 1.361***** | 0.715 | |
| Shannon | 0.181 | 0.908 | |  | | 0.797 | 0.511 | |  | | 1.782 | 0.185 | |  | | 0.351 | 0.789 | |
| Simpson | 0.371 | 0.775 | |  | | 0.964 | 0.430 | |  | | 1.593***** | 0.661 | |  | | 0.391 | 0.761 | |
| Chao1 | 2.507 | 0.090 | |  | | 0.320***** | 0.956 | |  | | 3.760 | **0.028** | |  | | 2.170***** | 0.538 | |
| ACE | 0.727***** | 0.867 | |  | | 0.290***** | 0.962 | |  | | 3.754 | **0.029** | |  | | 2.454***** | 0.484 | |
| Goods_coverage | 0.815***** | 0.846 | |  | | 0.020 | 0.996 | |  | | 0.970 | 0.427 | |  | | 0.090 | 0.965 | |
| Pielou | 0.176 | 0.911 | |  | | 0.555 | 0.651 | |  | | 4.517***** | 0.211 | |  | | 0.268 | 0.848 | |
| PD_whole_tree | 1.319 | 0.297 | |  | | 1.015 | 0.408 | |  | | 2.069 | 0.138 | |  | | 0.704***** | 0.872 | |

*****The data that failed for the testing of normality (Shapiro-Wilk test) and homogeneity of variance (Bartlett test) were further tested using nonparametric Scheirer-Ray-Hare test

F: Two-way ANOVA; H*****: Scheirer-Ray-Hare test

Time represents two seasons: monsoon and intermonsoon

Space indicates the region located near 5°S, 0°, 5°N~10.5°N and 15°N, respectively

All, whole bacterial community; CRT, conditionally rare taxa; ART, always rare taxa; AT, abundant taxa

Bold font indicates significant value (*P* < 0.05)

**Table S3** Analysis of similarity (ANOSIM) statistics depicting the differences of bacterial community groupings at spatiotemporal scales

|  | All | |  | CRT | |  | ART | |  | AT | |
| --- | --- | --- | --- | --- | --- | --- | --- | --- | --- | --- | --- |
|  | R | P |  | R | P |  | R | P |  | R | P |
| Time |  |  |  |  |  |  |  |  |  |  |  |
| W/S | 0.710 | **0.001** |  | 0.996 | **0.001** |  | 0.975 | **0.001** |  | 0.576 | **0.001** |
| Space |  |  |  |  |  |  |  |  |  |  |  |
| S1 /S0 | -0.190 | 0.652 |  | 0.207 | 0.271 |  | 0.379 | 0.125 |  | -0.246 | 0.849 |
| S1 /S2 | -0.272 | 0.882 |  | -0.198 | 0.883 |  | 0.159 | 0.314 |  | -0.216 | 0.802 |
| S0 /S2 | 0.500 | 0.333 |  | 0.000 | 0.667 |  | 0.000 | 1.000 |  | 0.500 | 0.333 |
| S3 /S2 | 0.033 | 0.329 |  | 0.070 | 0.239 |  | 0.202 | 0.060 |  | 0.024 | 0.341 |

An R-statistic less than 0 represents complete random grouping

Time represents two seasons: monsoon and intermonsoon

Space indicates the region located near 5°S, 0°, 5°N~10.5°N and 15°N, respectively

All, whole bacterial community; CRT, conditionally rare taxa; ART, always rare taxa; AT, abundant taxa

Bold font indicates significant value (*P* < 0.05)

**Table S4** The captured significant variable and corresponding R^2^ of forward selection of environmental, spatial and temporal effects based on constrained analysis of principal coordinates model in variation partitioning analysis (VPA)

| Microbial taxa | Environmental | |  | Spatial | |  | Temporal | |
| --- | --- | --- | --- | --- | --- | --- | --- | --- |
|  | Variable | R^2^ |  | Variable | R^2^ |  | Variable | R^2^ |
| All | Temperature | 0.270 |  | PCNM1 + PCNM6 + PCNM2 | 0.157 |  | Time | 0.330 |
| CRT | Temperature | 0.253 |  | PCNM1 + PCNM6 | 0.158 |  | Time | 0.253 |
| ART | Temperature | 0.114 |  | PCNM1 + PCNM6 | 0.071 |  | Time | 0.118 |
| AT | Temperature | 0.250 |  | PCNM1 + PCNM8 | 0.167 |  | Time | 0.252 |

Spatial indicates the variables derived from spatial coordinates using principal coordinates of neighbour matrices (PCNM) analysis

Time represents the sampling date (square root of day) of monsoon and intermonsoon

**Table S5** The topological features of co-occurrence subnetworks of microbial community in the monsoon and intermonsoon

|  | Total | Monsoon | Intermonsoon | RT | AT |
| --- | --- | --- | --- | --- | --- |
| Edges^a^ | 7045 | 939 | 4431 | 5845 | 132 |
| Nodes^b^ | 542 | 234 | 277 | 483 | 44 |
| Average degree^c^ | 25.996 | 8.130 | 32.109 | 24.203 | 6.000 |
| Connectance^d^ | 0.048 | 0.035 | 0.117 | 0.050 | 0.140 |
| Diameter^e^ | 9.110 | 9.235 | 6.640 | 9.182 | 5.205 |
| Modularity^f^ | 0.274 | 0.446 | 0.238 | 0.264 | 0.418 |
| Average path length^g^ | 3.156 | 3.571 | 2.643 | 3.205 | 2.538 |
| Clustering coefficient^h^ | 0.481 | 0.343 | 0.577 | 0.497 | 0.518 |
| Average path length^i^ ± SD | 2.555 ± 0.021 | 2.946 ± 0.028 | 2.218 ± 0.031 | 2.568 ± 0.021 | 2.489 ± 0.072 |
| Clustering coefficient^j^ ± SD | 0.207 ± 0.009 | 0.107 ± 0.009 | 0.275 ± 0.01 | 0.214 ± 0.009 | 0.204 ± 0.027 |

^a^ Number of edges

^b^ Number of nodes

^c^ Average degree; shows the average of how many connections each node has to another unique node in the network

^d^ Graph density; The intensity of connections among nodes

^e^ The length of the longest geodesic between the nodes that exists in the network

^f^ Modularity >0.4 indicates that the network has a modular structure. A highly modularized network suggests that there are nodes in the network that are more densely connected between each other than with the rest of the network.

^g^ The length of all the shortest paths from or to the nodes in the network

^h^ Transitivity; measures the probability that the adjacent nodes of a vertex are connected (Barr*at et a*l., 2004, Newman, 2006, Scott, 2016)

^i^ The average length of all the shortest paths from or to the nodes in the random network

^j^ Transitivity; measures the probability that the adjacent nodes of a vertex are connected in the random network

**Table S6** Pearson’s correlations of the bacterial community with environmental factors based on Mantel tests

|  | | All | CRT | ART | AT | Network |
| --- | --- | --- | --- | --- | --- | --- |
| Time | r | 0.686 | 0.804 | 0.800 | 0.605 | 0.703 |
|  | p | **0.001** | **0.001** | **0.001** | **0.001** | **0.001** |
| Temperature | r | 0.524 | 0.603 | 0.568 | 0.462 | 0.515 |
|  | p | **0.001** | **0.001** | **0.001** | **0.001** | **0.001** |
| NO_2_^-^ | r | 0.355 | 0.384 | 0.398 | 0.328 | 0.388 |
|  | p | **0.001** | **0.001** | **0.001** | **0.001** | **0.001** |
| Chl *a* | r | 0.123 | 0.042 | 0.060 | 0.134 | 0.104 |
|  | p | 0.135 | 0.281 | 0.179 | 0.135 | 0.148 |
| P | r | 0.112 | 0.048 | 0.044 | 0.107 | 0.093 |
|  | p | 0.112 | 0.208 | 0.202 | 0.128 | 0.126 |
| NH_4_^+^ | r | -0.101 | 0.050 | 0.013 | -0.135 | -0.062 |
|  | p | 0.837 | 0.227 | 0.366 | 0.900 | 0.754 |
| NO_3_^-^ | r | -0.047 | 0.012 | 0.076 | -0.056 | -0.074 |
|  | p | 0.654 | 0.415 | 0.142 | 0.637 | 0.792 |
| Si | r | -0.071 | -0.016 | 0.005 | -0.081 | -0.066 |
|  | p | 0.767 | 0.576 | 0.434 | 0.766 | 0.783 |
| Salinity | r | -0.050 | 0.083 | 0.117 | 0.071 | -0.075 |
|  | p | 0.705 | 0.108 | **0.049** | 0.778 | 0.863 |

Time represents the sampling date (square root of day) of monsoon and intermonsoon

All, whole bacterial community; CRT, conditionally rare taxa; ART, always rare taxa; AT, abundant taxa

The significances are tested based on 999 permutations, and bold font indicates significant value (*P* < 0.05)

**Table S7 Pearson’s correlations of the relative abundance of different bacterial phyla with environmental factors based on Mantel tests**

| Relative abundance | Time | |  | Temperature | |  | NO_2_^-^ | |  | Chl *a* | |
| --- | --- | --- | --- | --- | --- | --- | --- | --- | --- | --- | --- |
|  | r | p |  | r | p |  | r | p |  | r | p |
| Acidobacteria | 0.082 | 0.133 |  | 0.152 | 0.099 |  | 0.026 | 0.391 |  | -0.201 | 0.961 |
| Actinobacteria | 0.328 | **0.001** |  | 0.284 | **0.005** |  | 0.057 | 0.263 |  | -0.122 | 0.834 |
| Bacteroidetes | 0.108 | 0.115 |  | -0.011 | 0.418 |  | 0.183 | **0.042** |  | -0.106 | 0.741 |
| Chloroflexi | 0.085 | 0.139 |  | 0.107 | 0.168 |  | -0.092 | 0.806 |  | -0.061 | 0.533 |
| Cyanobacteria | 0.246 | **0.003** |  | 0.192 | **0.035** |  | 0.145 | 0.072 |  | 0.200 | 0.096 |
| Firmicutes | 0.082 | 0.132 |  | 0.143 | 0.101 |  | -0.078 | 0.732 |  | -0.053 | 0.460 |
| Fusobacteria | 0.124 | 0.059 |  | 0.101 | 0.165 |  | -0.096 | 0.812 |  | -0.056 | 0.515 |
| Gemmatimonadetes | 0.189 | **0.017** |  | 0.270 | **0.008** |  | 0.008 | 0.438 |  | -0.207 | 0.955 |
| Gracilibacteria | 0.147 | **0.047** |  | 0.070 | 0.249 |  | 0.004 | 0.440 |  | -0.001 | 0.380 |
| Nitrospirae | 0.157 | 0.055 |  | 0.079 | 0.251 |  | 0.040 | 0.347 |  | -0.185 | 0.956 |
| Others | 0.241 | **0.002** |  | 0.142 | 0.109 |  | 0.108 | 0.123 |  | -0.057 | 0.530 |
| Planctomycetes | 0.107 | 0.088 |  | 0.164 | 0.075 |  | -0.017 | 0.538 |  | 0.057 | 0.311 |
| Proteobacteria | 0.455 | **0.001** |  | 0.344 | **0.001** |  | 0.423 | **0.001** |  | 0.098 | 0.192 |
| Saccharibacteria | 0.081 | 0.121 |  | 0.195 | 0.063 |  | -0.036 | 0.613 |  | -0.162 | 0.878 |
| SAR406_clade | 0.024 | 0.281 |  | 0.032 | 0.327 |  | 0.025 | 0.317 |  | 0.037 | 0.290 |
| SBR1093 | 0.511 | **0.001** |  | 0.243 | **0.004** |  | 0.427 | **0.001** |  | 0.052 | 0.272 |
| Tenericutes | 0.108 | 0.076 |  | 0.250 | **0.033** |  | 0.001 | 0.427 |  | -0.072 | 0.559 |
| Verrucomicrobia | 0.180 | **0.017** |  | 0.043 | 0.316 |  | 0.048 | 0.295 |  | -0.097 | 0.650 |

**Table S7 Continued**

| Relative abundance | P | |  | NH_4_^+^ | |  | NO_3_^-^ | |  | Si | |  | Salinity | |
| --- | --- | --- | --- | --- | --- | --- | --- | --- | --- | --- | --- | --- | --- | --- |
|  | r | p |  | r | p |  | r | p |  | r | p |  | r | p |
| Acidobacteria | -0.053 | 0.593 |  | 0.034 | 0.377 |  | -0.182 | 0.903 |  | -0.022 | 0.453 |  | 0.143 | 0.148 |
| Actinobacteria | 0.121 | 0.171 |  | -0.051 | 0.538 |  | -0.094 | 0.754 |  | -0.039 | 0.558 |  | -0.061 | 0.616 |
| Bacteroidetes | -0.065 | 0.592 |  | -0.109 | 0.773 |  | -0.003 | 0.293 |  | -0.109 | 0.855 |  | -0.077 | 0.641 |
| Chloroflexi | 0.278 | 0.051 |  | -0.008 | 0.391 |  | 0.020 | 0.277 |  | 0.123 | 0.145 |  | 0.064 | 0.273 |
| Cyanobacteria | 0.144 | 0.118 |  | -0.235 | 0.995 |  | -0.092 | 0.685 |  | -0.115 | 0.836 |  | -0.012 | 0.523 |
| Firmicutes | 0.275 | 0.060 |  | -0.041 | 0.476 |  | 0.036 | 0.208 |  | 0.015 | 0.291 |  | -0.031 | 0.469 |
| Fusobacteria | 0.119 | 0.209 |  | -0.061 | 0.587 |  | 0.027 | 0.304 |  | 0.057 | 0.254 |  | 0.244 | **0.038** |
| Gemmatimonadetes | 0.079 | 0.246 |  | 0.180 | 0.110 |  | -0.166 | 0.859 |  | 0.083 | 0.246 |  | 0.174 | 0.087 |
| Gracilibacteria | 0.209 | 0.074 |  | -0.048 | 0.545 |  | 0.494 | **0.016** |  | 0.022 | 0.315 |  | -0.021 | 0.519 |
| Nitrospirae | -0.061 | 0.638 |  | 0.151 | 0.157 |  | -0.180 | 0.935 |  | 0.016 | 0.363 |  | -0.023 | 0.521 |
| Others | -0.060 | 0.633 |  | 0.014 | 0.375 |  | -0.102 | 0.714 |  | -0.106 | 0.814 |  | -0.095 | 0.775 |
| Planctomycetes | 0.087 | 0.270 |  | -0.066 | 0.627 |  | 0.029 | 0.299 |  | 0.157 | 0.106 |  | 0.090 | 0.247 |
| Proteobacteria | -0.086 | 0.819 |  | -0.018 | 0.543 |  | -0.119 | 0.853 |  | 0.084 | 0.217 |  | -0.009 | 0.539 |
| Saccharibacteria | 0.230 | 0.053 |  | 0.044 | 0.343 |  | -0.127 | 0.742 |  | 0.160 | 0.121 |  | 0.131 | 0.151 |
| SAR406_clade | -0.009 | 0.482 |  | -0.030 | 0.534 |  | -0.055 | 0.632 |  | 0.133 | 0.122 |  | -0.011 | 0.502 |
| SBR1093 | 0.181 | **0.040** |  | -0.094 | 0.825 |  | 0.017 | 0.366 |  | -0.093 | 0.840 |  | -0.105 | 0.886 |
| Tenericutes | 0.020 | 0.358 |  | 0.023 | 0.338 |  | 0.049 | 0.278 |  | 0.117 | 0.152 |  | 0.129 | 0.142 |
| Verrucomicrobia | 0.190 | 0.083 |  | 0.131 | 0.177 |  | -0.144 | 0.801 |  | -0.100 | 0.724 |  | -0.132 | 0.877 |

**References**

Barrat A, Barthelemy M, Pastor-Satorras R & Vespignani A (2004) The architecture of complex weighted networks. *Proc Natl Acad Sci U S A* **101**: 3747-3752.

Clarke KR (1993) Non-parametric multivariate analyses of changes in community structure. *Austral Ecology* **18**: 117-143.

Field A, Miles J & Field Z (2012) *Discovering statistics using R*. Sage publications.

Newman ME (2006) Modularity and community structure in networks. *Proc Natl Acad Sci U S A* **103**: 8577-8582.

Oksanen J, Blanchet FG, Kindt R, Legendre P, O’hara R, Simpson GL, Solymos P, Stevens MHH & Wagner HJUhCR-popv (2010) Vegan: community ecology package. R package version 1.17-4.

Scott J (2016) Social Network Analysis. *Sociology* **22**: 109-127.

Shiozaki T, Kodama T, Kitajima S, Sato M & Furuya K (2013) Advective transport of diazotrophs and importance of their nitrogen fixation on new and primary production in the western Pacific warm pool. *Limnology and Oceanography* **58**: 49-60.

Sokal RR (1995) The principles and practice of statistics in biological research. *Biometry* 451-554.
